# Comparative multi-omics systems analysis of *Escherichia coli *strains B and K-12

**DOI:** 10.1186/gb-2012-13-5-r37

**Published:** 2012-05-25

**Authors:** Sung Ho Yoon, Mee-Jung Han, Haeyoung Jeong, Choong Hoon Lee, Xiao-Xia Xia, Dae-Hee Lee, Ji Hoon Shim, Sang Yup Lee, Tae Kwang Oh, Jihyun F Kim

**Affiliations:** 1 Systems and Synthetic Biology Research Center, Korea Research Institute of Bioscience and Biotechnology, Yuseong, Daejeon 305-806, Korea; 2 Metabolic and Biomolecular Engineering National Research Laboratory, Department of Chemical and Biomolecular Engineering, BioProcess Engineering Research Center, Center for Systems and Synthetic Biotechnology, and Institute for the BioCentury, Korea Advanced Institute of Science and Technology, Yuseong, Daejeon 305-701, Korea; 3 Department of Biomolecular and Chemical Engineering, Dongyang University, Yeongju, Gyeongbuk, 750-711, Korea; 4 Department of Biological Sciences, Korea Advanced Institute of Science and Technology, Yuseong, Daejeon 305-701, Korea; 5 Department of Systems Biology, Yonsei University, 50 Yonsei-ro, Seodaemun-gu, Seoul 120-749, Korea; 6 Department of Bio and Brain Engineering, and Bioinformatics Research Center, Korea Advanced Institute of Science and Technology, Yuseong, Daejeon 305-701, Korea; 7 21C Frontier Microbial Genomics and Applications Center, Korea Research Institute of Bioscience and Biotechnology, Yuseong, Daejeon 305-806, Korea

## Abstract

**Background:**

Elucidation of a genotype-phenotype relationship is critical to understand an organism at the whole-system level. Here, we demonstrate that comparative analyses of multi-omics data combined with a computational modeling approach provide a framework for elucidating the phenotypic characteristics of organisms whose genomes are sequenced.

**Results:**

We present a comprehensive analysis of genome-wide measurements incorporating multifaceted holistic data - genome, transcriptome, proteome, and phenome - to determine the differences between *Escherichia coli *B and K-12 strains. A genome-scale metabolic network of *E. coli *B was reconstructed and used to identify genetic bases of the phenotypes unique to B compared with K-12 through *in silico *complementation testing. This systems analysis revealed that *E. coli *B is well-suited for production of recombinant proteins due to a greater capacity for amino acid biosynthesis, fewer proteases, and lack of flagella. Furthermore, *E. coli *B has an additional type II secretion system and a different cell wall and outer membrane composition predicted to be more favorable for protein secretion. In contrast, *E. coli *K-12 showed a higher expression of heat shock genes and was less susceptible to certain stress conditions.

**Conclusions:**

This integrative systems approach provides a high-resolution system-wide view and insights into why two closely related strains of *E. coli*, B and K-12, manifest distinct phenotypes. Therefore, systematic understanding of cellular physiology and metabolism of the strains is essential not only to determine culture conditions but also to design recombinant hosts.

## Background

*Escherichia coli *is one of the most intensively studied organisms and has been widely employed in scientific studies and industrial applications. The most widely used *E. coli *have been those derived from strains B and K-12, the result of pioneering work using K-12 for genetic and biochemical studies and B for studying virulent bacteriophages, restriction systems, mutagenic assays, and bacterial evolution [1,2]. The first whole-genome sequence of strain K-12, MG1655, was determined [3] and compared in detail with another K-12 strain, W3110 [4]. We have determined genome sequences of B strains [5,6] - REL606, which has been applied to the study of long-term experimental evolution, and BL21(DE3), which has been used as a cell-factory for overproducing recombinant proteins, biofuels, and a variety of bioproducts on an industrial scale.

Previously, we showed that comparison of the genome sequences of strains B and K-12 could provide plausible explanations for some long-known differences between them [7]. However, genome sequence alone provides limited information about the genotype-phenotype relationship [8]. Starting with a genome sequence, comprehensive analyses of transcriptomes, proteomes, and phenomes can complement each other and be integrated to provide deeper insights into biological systems [9-12]. Furthermore, the multidimensional omics data can be integrated to reconstruct genome-wide computational models that can generate testable hypotheses concerning cellular function [13,14].

Here, we systematically combined the results of comparative analyses of the genomes, transcriptomes, proteomes, and phenomes of *E. coli *B REL606 and K-12 MG1655 to decipher the whole-organism characteristics that differentiate these two strains. An *in silico *genome-scale metabolic model of *E. coli *B REL606 was reconstructed and used to determine the genetic basis of the phenotypic differences.

## Results

### Genomic differences

**Figure 1:**
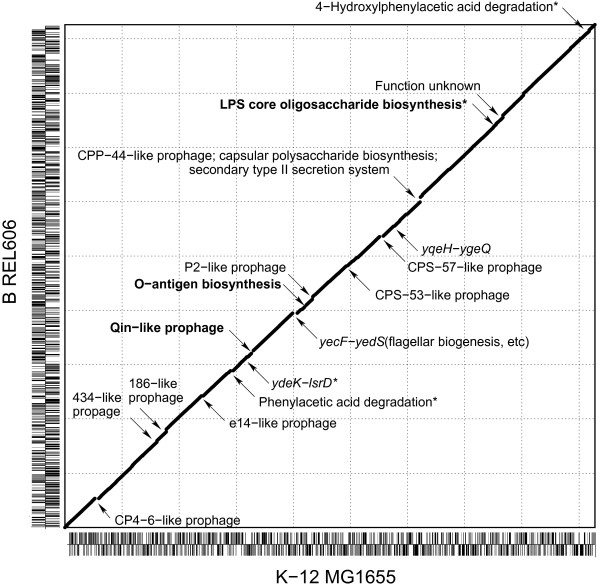
**Whole genome comparison of *E. coli *B REL606 and *E. coli *K-12 MG1655**. Strain-specific regions are indicated by discontinuities on the diagonal line (those >10 kb are marked by arrows). Short vertical lines on each axis represent coding sequences that reside on the forward or reverse strand. Segments that occupy the same location on each genome and encode equivalent functions but are highly dissimilar are shown in bold. Except for those marked with asterisks, all the strain-specific regions coincide with genomic islands that were identified by genomic anomalies. Ticks are marked every 500 kb.

The average nucleotide identity of the aligned genomic regions of REL606 [GenBank:NC_012967] [6] and MG1655 [GenBank:NC_000913] [3] was >99.1%. Only about 4% of the total genome accounts for strain-specific regions, including prophages and seemingly recently transferred genomic islands (Figure 1). Interestingly, the B genome encodes an additional set of genes for type II secretion (T2S) and D-arabinose utilization, and lacks the gene cluster for flagellar biosynthesis and the very short-patch repair system. Different sets of genes were observed for the Qin prophage, O antigen biosynthesis, catabolism of aromatic compounds, and lipopolysaccharide (LPS) oligosaccharide biosynthesis. Different gene clusters for the catabolism of aromatic compounds were also detected; the *hpa *cluster for degradation of 3- and 4-hydroxy phenyl acetic acid in the B strain and the *paa *cluster for catabolism of phenyl acetic acid in the K-12 strain. We found numerous strain-specific gene disruptions caused by deletions, frameshifts, or insertions of insertion sequence (IS) elements that might affect bacterial phenotypes (Table S1 in Additional file 1).

### Bacterial cell growth

Two *E. coli *B strains [6], REL606 and BL21(DE3), and two K-12 strains [4], MG1655 and W3110, were cultured in Luria-Bertani (LB) medium or R/2 medium (Figure S1 in Additional file 1). Both groups of strains grew similarly when cultured in complex medium; however, the B strains grew faster than the K12 strains in minimal medium. The difference between the strain types in the accumulation of by-products such as acetic, formic, and lactic acids was negligible when cultured in complex medium compared with growth in minimal media.

### Transcriptomic differences

**Figure 2:**
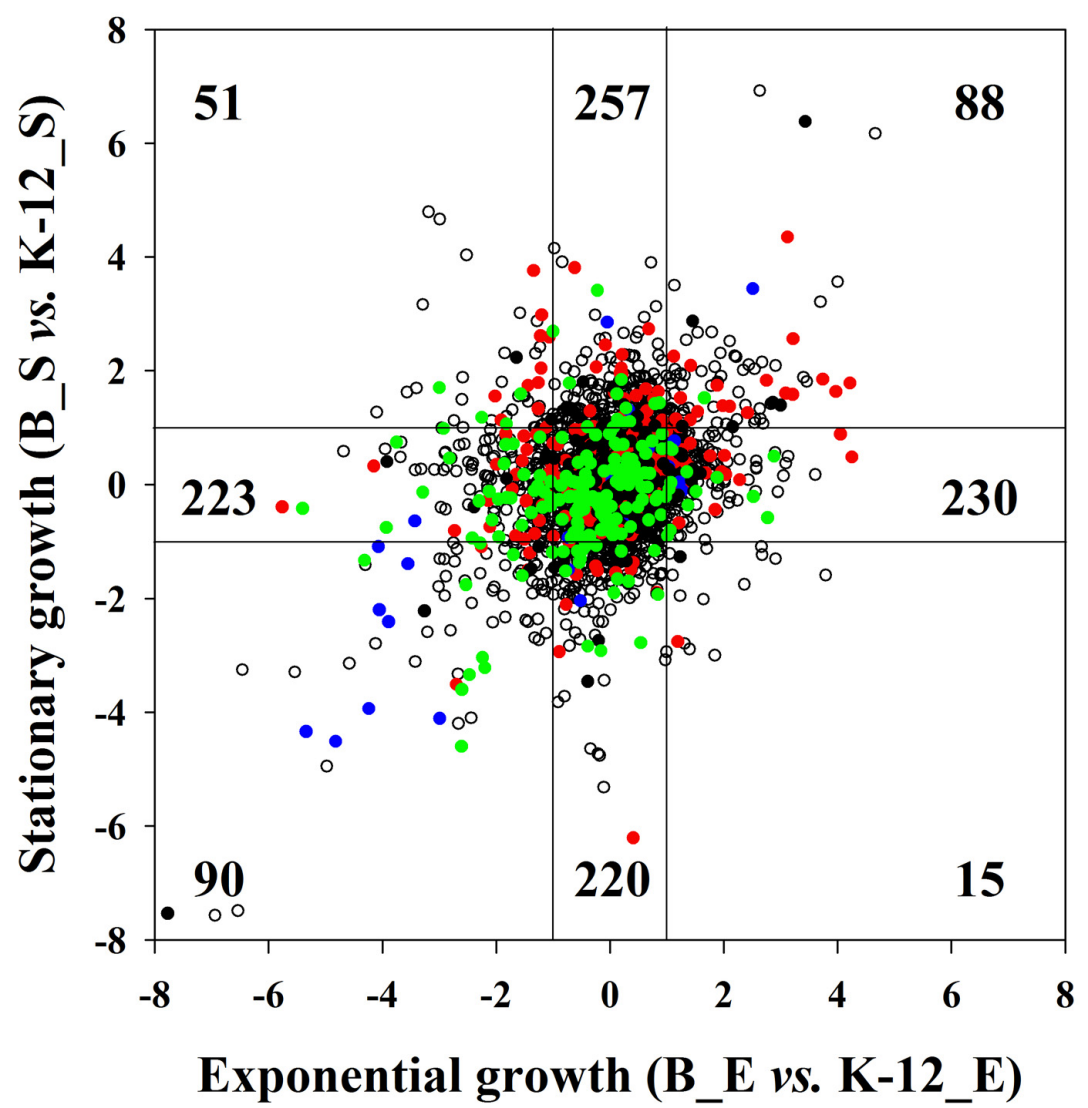
**Transcription ratios of *E. coli *B REL606 to K-12 MG1655 at exponential (E) and stationary (S) growth phases during growth in LB medium**. Internal lines were positioned at ±1.0 on both axes denoting log_2_-transformed expression ratios. The numbers of genes within each range of expression ratios are shown. A functional category using the COG database [45] was assigned to each gene and color-coding was employed as follows: red circles, amino acid transport and metabolism; blue circles, cell motility; green circles, energy production and conversion; black circles, biogenesis of cell wall components; empty circles, other or unknown function.

Transcriptome profiles were analyzed using samples taken in the exponential and stationary growth phases during cultivation of REL606 and MG1655 in LB medium. During the exponential growth phase, the most highly expressed genes in the B strain were those involved in replication, translation, or nucleotide transport and metabolism, while many of the highly expressed genes in the K-12 strain were involved in cell motility, transcription, carbohydrate transport, or energy production (Figure 2; Table S2 in Additional file 1). At the stationary phase, many of the genes that were highly expressed in the B strain were involved in transport and metabolism of various amino acids and carbohydrates, whereas highly expressed genes in the K-12 strain had functions related to cell motility, ribosomal subunit protein production, or energy generation.

Many genes showed highly distinct expression levels in REL606 and MG1655 irrespective of growth conditions (upper right for REL606 and lower left for MG1655 in Figure 2; also see Table S2 in Additional file 1). Highly expressed genes in REL606 included those encoding enzymes for biosynthesis of L-arginine (*argAGDECBHI*) and branched-chain amino acids (*ilvGMEDA*), a subunit of the L-arginine transporter (*artJ*), cytochrome b562 (*cybC*), subunits of the histidine ABC transporter (*hisPJ*), cytotoxins (*hokED*), outer membrane porin (*ompF*), L-arginine decarboxylase (*speA*), and a cell division inhibitor (*sulA*). In MG1655, highly expressed genes included those involved in chemotaxis (*cheZYRWA*, *tap*, *trg*, *tsr*), Lon protease (*lon*), C_4_-dicarboxylate-sensing histidine kinase (*dcuS*), chaperones (*clpB*, *dnaK*, *groES*, *htpG*, *ibpA*), the major subunit of type 1 fimbriae (*fimA*), a regulator of flagellar biosynthesis (*flhC*), glycerol-3-phosphate-dehydrogenase (*glpABCD*), glycerophosphoryl diester phosphodiesterase (*glpQ*), glycerol-3-phosphate transporter (*glpT*), hydrogenase 2 (*hybCBO*), outer membrane porins (*nmpC*, *ompA*, *ompC*), and galactitol transport and metabolism (gatYZC).

### Proteomic differences

Proteome analyses of the total intracellular, outer membrane, and extracellular proteomes of the four *E. coli *strains cultured in LB medium (Figure S2 in Additional file 1) revealed 18 protein spots of B strains and 42 spots of K-12 strains that showed a more than two-fold difference in intensity and the corresponding proteins were further characterized (Table S3 in Additional file 1). Intracellular proteins that were more abundant in the B strains were enzymes required for the biosynthesis of some amino acids (AspC, ArgCDI, SerC) and maltose metabolism (MalPQ). Proteins that were more abundant in K-12 strains included enzymes for the transport and metabolism of galactitol (GatYZAB), enzymes for the degradation of amino acids (TdcE, TnaA), and stress response proteins (ClpP, CspE, YfiD). The proteome profiles of the two B strains were very similar, as were those of the two K-12 strains (Figure S2A in Additional file 1). Proteins involved in chemotaxis (CheY) and a degradation pathway for amino acids (TdcF) were synthesized at a significant level only by MG1655.

SDS-PAGE analysis of outer membrane proteins (OMPs) revealed that the B strains expressed large amounts of OmpF but not OmpC, whereas K-12 strains expressed both OmpF and OmpC (Figure S2B in Additional file 1). The expression level of OmpA was higher in K-12 strains than in B strains.

**Figure 3:**
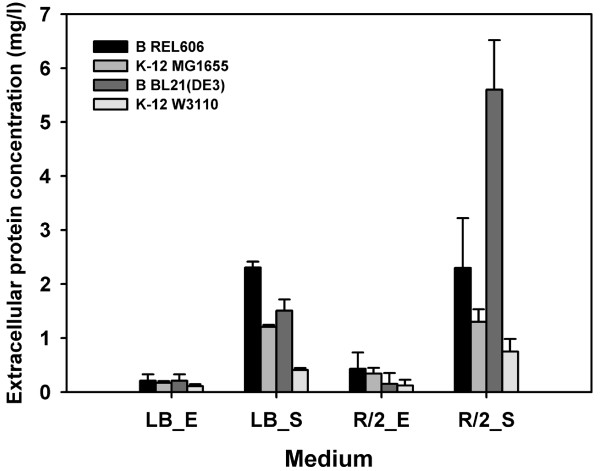
**Extracellular protein concentrations of *E. coli *B and *E. coli *K-12**. Extracellular proteins were precipitated from the supernatants of each *E. coli *strain grown in LB and minimal R/2 media at the exponential (E) and stationary (S) growth phases.

**Figure 4:**
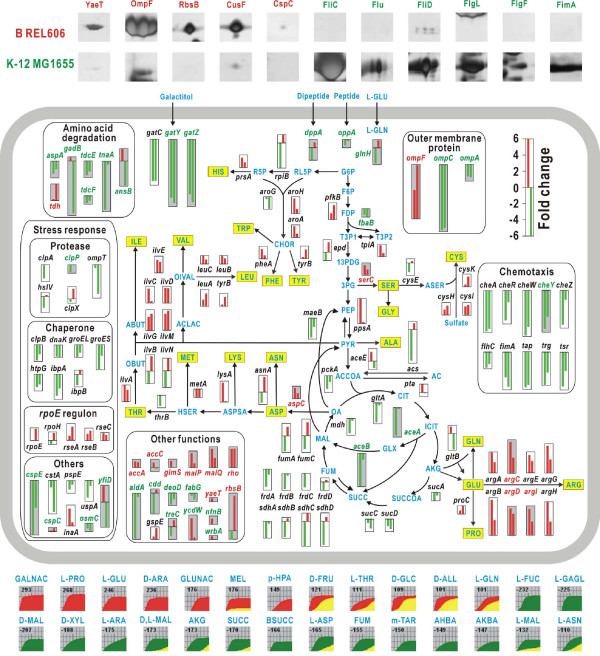
**Comparison of the transcriptomes, proteomes, and phenomes of *E. coli *B REL606 and *E. coli *K-12 MG1655 cultured in LB medium**. In the individual thumbnail graphs, the x-axes denote exponential and stationary growth phases, and the y-axes show the transcription ratio (on a log_2 _scale) of each gene in the B strain to that of the homologous gene in the K-12 strain. The transcription bars are coded red for up-regulation in B and green for up-regulation in K-12. Genes showing differences in transcriptional (or translational) levels of ≥2- or ≤0.5-fold in the exponential and/or stationary phase(s) are shown. The backgrounds of the graphs are grey if both transcriptional and translational differences were detected. Gene names are shown in red if the translational level was higher in the B strain and green if it was higher in K-12. The top of the figure shows extracellular proteins with more than a two-fold difference between the strains in the stationary phase on growth in minimal medium. Significant differences in cell growth on particular carbon sources, demonstrated by phenotype microarray tests, are shown at the bottom. Growth curves are green for B, red for K-12, and yellow for the overlapping region. Abbreviations are given in Additional file 1.

Examination of the extracellular proteome (Figure S2C in Additional file 1) revealed that B strains released larger amounts of protein in the stationary growth phase than K-12 strains (Figure 3). Proteins commonly released from both B and K-12 strains were outer membrane proteins (OmpA, OmpF, and OmpX) and transport/binding proteins (ArgT, ArtI, ArtJ, CusF, GlnH, HisJ, and LivJ). The outer membrane proteins OmpF and YaeT, the periplasmic substrate transport and binding proteins CusF and RbsB, and the cold shock protein CspC were released at higher levels from B than from K-12 strains, whereas motility-related proteins (FimA, FlgF, FlgL, FliC, and FliD) and Flu were secreted only by K-12 strains (top of Figure 4).

### Phenomic differences

Phenotype microarray (PM) tests on REL606 and MG1655 revealed dramatic phenotypic differences between the two strains (Figure S3 in Additional file 1). The B strain was more susceptible than the K-12 strain to a variety of stressful conditions caused by osmolarity, pH, or exposure to inhibitory compounds such as salicylate and β-lactam antibiotics. MG1655 did not grow on valine dipeptides.

### Reconstruction of the metabolic network

We reconstructed a genome-scale metabolic network model of REL606 by incorporating genetic differences between REL606 and MG1655 (Table S4 in Additional file 1). Starting with the metabolic network model for MG1655 (iAF1260) [15], 29 REL606-specific reactions and 11 REL606-specific compounds were added, 12 REL606-specific regulations were introduced, and 43 MG1655-specific reactions were excluded. The resulting metabolic model of REL606 included 1,369 metabolic reactions and 1,051 metabolites. To validate the model, flux balance analysis (FBA) was used to predict cell growth on various carbon sources, and the results were compared with the results from PM tests (Table S5 in Additional file 1). The model qualitatively agreed with 83.2% of experimental cell growth results on PM plates testing carbon source utilization.

## Discussion

### Lower acetate accumulation of the B strain might be attributed to different regulation of the glyoxylate pathway

Acetate accumulation is one of the major problems encountered during high cell density cultivation of *E. coli *because it inhibits cell growth and production of foreign proteins [16]. Low acetate accumulation by B strains is commonly attributed to the active glyoxylate shunt encoded by the acetate operon (*aceBAK*); however, in the exponential growth stage, up-regulation of genes involved in the glyoxylate shunt, tricarboxylic acid (TCA) cycle cycle, and gluconeogenesis was observed in K-12 strains (Figure 4). These expression patterns are similar to results from previous microarray analysis comparing transcriptomes of K-12 JM109 and BL21 during low glucose-fed batch fermentation [17]. The need for high activity of the TCA cycle in K-12 strains cultured in low glucose media is not immediately obvious, but enhanced catabolic activity may be required for the energy-consuming motility and stress responses (see below).

Genes involved in the central metabolism and the acetate simulation/dissimilation pathways (glycolysis, TCA cycle, pentose phosphate pathway, glyoxylate pathway, gluconeogenesis, and acetate production) were virtually the same at the nucleotide level in B and K-12 strains. This was also the case for the transcriptional regulator gene *fruR*, which was suspected to have altered expression or regulatory strength in BL21, leading to derepression of acetate metabolism even under high glucose culture conditions [17]. Interestingly, *arpA*, which has an unknown function but has been proposed to play a role in regulation of the glyoxylate pathway [18], is disrupted in B strains.

Many of the phenotypic differences between B and K-12 strains in utilization of carbon sources, as shown in PM tests (Figure S3 in Additional file 1), could be correlated with genotypic differences (Table S5 in Additional file 1). In particular, genes for galactitol transport and metabolism were highly expressed at both the mRNA level (*gatYZC*) and the protein level (GatYZAB) in MG1655, possibly because *gatR*, a repressor of the *gat *genes, is truncated in MG1655 but intact in REL606.

### Enhanced amino acid biosynthesis and reduced degradation can make the B strain favorable for efficient production of recombinant proteins

**Figure 5:**
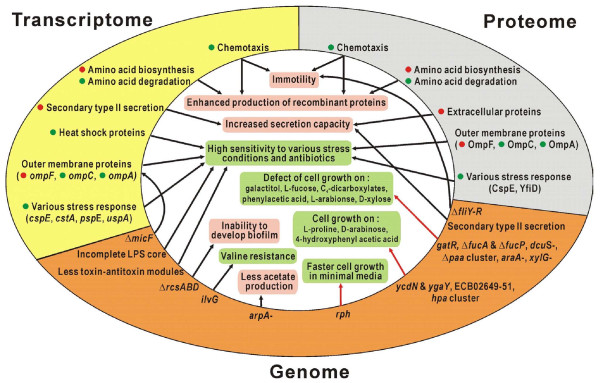
**Proposed explanations for the observed and predicted phenotypes of *E. coli *B strains derived from the combined analysis of genome, transcriptome, and proteome data**. In the inner circle, observations from this study are marked with a green background and hypotheses generated in this study are shown in pink. Genes with elevated mRNA or protein expression in B (red circle) or K-12 (green circle) strains are shown. Genetic differences between B and K-12 strains are denoted as follows: Δ, absence in B; -, pseudogene in B. Red arrows denote the phenotypes and related genetic backgrounds supported by flux balance analysis using the reconstructed metabolic model of the B strain.

Major differences were observed in the expression of genes involved in the biosynthesis and degradation of amino acids (Figure 4). Most of the genes involved in biosynthetic pathways of amino acids, especially those required for biosynthesis of arginine and branched-chain amino acids (leucine, isoleucine, and valine), were more highly expressed in the B strain than in K-12. Genes encoding the three isozymes of acetohydroxy acid synthase I (*ilvBN*), II (*ilvGM*) and III (*ilvIH*) exhibited complicated expression patterns (Figure 4), which may be attributed to transcriptional control by attenuation [19]. A frameshift mutation was found in the *ilvG *gene of the K-12 strains, whereas this gene is intact in the B strains. The gene *ilvG *mediates valine resistance and allows growth on various valine dipeptides in PM tests (Figure S3 in Additional file 1); this explains the failure of MG1655 to grow on valine dipeptides. Proteome analysis also indicated that enzymes required for the biosynthesis of arginine (ArgDCI), serine (SerC), and aspartate (AspC) were synthesized at high levels in the B strains. Interestingly, TnaA, a protein involved in the tryptophan and cysteine degradation pathway, was highly expressed at both the mRNA and protein levels in the K-12 strains. The same was true for TdcE and TdcF, required for anaerobic degradation of L-threonine, GadB, involved in glutamate degradation and acid resistance, and AspA, needed for the reversible conversion of L-aspartate to fumarate and ammonia. These observations suggest that the B strains are, in general, disposed to enhanced amino acid biosynthesis and reduced degradation, conditions favorable for efficient production of recombinant proteins (Figure 5).

### Lack of flagellar biosynthetic genes and lower expression of motility genes are important properties of strain B as a protein factory

The cell envelope is the principal stress-bearing and shape maintenance element in *E. coli*, and its integrity is critical for cell viability. B strains have been widely used for mutagenic assays and toxicological studies because they show higher membrane permeability than K-12 strains [20]. Structural studies on the LPS core oligosaccharide have revealed that K-12 strains are devoid of the O antigen while B strains lack the O antigen and the distal part of the core [21]. Sequence comparison revealed that the outer membrane structure of B strains is quite different from that of K-12 strains [6]. In both groups, IS elements are inserted at the gene clusters for O antigen biosynthesis: at *wbbL *for K-12 strains and between *manC *and *wbbD *for B strains. In the genome of B strains, the core part of LPS was further disrupted by the insertion of IS*1 *at the *waaT *gene encoding UDP-galatose:(glucosyl) LPS α1,2-galactosyltransferase. The B strain showed high expression of *glmS*, encoding a protein involved in both cell wall and LPS biosynthesis [22], at both the mRNA and protein level (Figure 4).

Unexpectedly, flagellar biosynthesis genes (*fliY-R*) were absent from the B genome, rendering B strains non-motile [6]. This agrees with microarray data that indicated that expression levels of chemotaxis genes (*cheZYRWA*, *tap*, *trg*, and *tsr*) and of *flhC*, encoding the master regulator of flagellar biosynthesis, are much lower in B than in K-12 (Figure 4). This finding is also concordant with the proteomic data showing CheY, FimA, FlgF, FlgL, FliC, and FliD only in K-12 strains. Lack of flagellar biosynthetic genes and low expression of motility-related genes might be important properties when B strains are used as protein factories, because flagellar biosynthesis is energy-intensive and is not necessary under industrial conditions of constant agitation and generous nutrient supply [23].

### Different cellular responses to environmental changes were observed for B and K-12 strains

The susceptibility of the *E. coli *B strain to a variety of stressful conditions and antibiotics revealed by PM tests (Figure S3 in Additional file 1) can be explained by several observations (Figure 5). First, differences in the composition of the LPS core and expression of outer membrane proteins may influence the permeability and integrity of the cell envelope. B strains produce more OmpF porin than K-12 strains because the B genome lacks *micF*, which post-transcriptionally prevents production of OmpF [24]. This is further supported by the transcriptome data showing high levels of *ompF *expression in the B strain and high expression of *ompC *and *ompA *in the K-12 strain (Figure 4). These observations were also consistent with results of proteome analysis of the outer membrane fractions (Figure S2B in Additional file 1). Noxious agents such as antibiotics and bile acids diffuse more easily through OmpF than OmpC because the former produces a channel with a larger pore size [25]. Second, synthesis of capsular colanic acid and biofilm formation appear to be repressed in B strains as a result of the absence of the Rcs phosphorelay system (*rcsADB*), the major mechanism regulating these processes [26]. Proteome data also showed that FimA and Flu involved in biofilm formation are secreted only by K-12 strains. Third, genome sequence comparisons revealed at least five toxin-antitoxin (TA) modules in K-12, three *relBE-*class loci (*relBE*, *dinJ*-*yafQ *and *yefM*-*yoeB*) and two *mazEF*-homologous loci (*chpRA *and *chpSB*), whereas B strains possess only two TA loci (*dinJ*-*yafQ*, *chpRA*). TA modules play an important role in bacterial stress physiology and might contribute to multidrug resistance [27]. Finally, the B strain showed lower stress responses. Heat shock genes encoding molecular chaperones (*dnaK*, *groES*, *htpG*, *ibpA*, and *clpB*) and ATP-dependent proteases (*clpA*, *clpP*, and *hslV*) were expressed at lower levels in the B strain (Figure 4). Furthermore, B strains are naturally deficient in the major protease Lon as a result of an IS*186 *insertion in the promoter region [28]. Expression levels of DnaK and ClpB, which correlate with cellular stress levels, are lower in BL21 strains than in K-12 strains (JM105, HB101, and TOP10) [29]. K-12 also showed activation of genes for cold shock protein (*cspE*), carbon starvation protein (*cstA*), osmotically inducible peroxidase (*osmC*), phage shock protein (*pspE*), and universal stress protein (*uspA*) (Figure 4), which may make K-12 strains more resistant than B strains to various stress conditions.

The *rpoE *gene, encoding heat-inducible sigma factor σ^E^, and some genes in the operon (*rseABC*) regulated by this sigma factor, were highly expressed in REL606 (Figure 4). σ^E ^induces an extracytoplasmic stress response to misfolded outer membrane proteins and damage to the cell envelope [30]. Variations in expression of *rpoE *regulon genes might result from differences in outer membrane composition between the two strain types.

### Expression of the additional T2S genes of the B strain might be responsible for its larger amounts of secreted proteins

Interestingly, the B genome has an additional gene cluster for T2S that consists of the homologs *gspOCDEFGHIJKLM*. From a phylogenetic analysis of the concatenated sequences of GspDEFG, clear distinctions could be made between homologs of T2S that were common to B and K-12 and the additional T2S genes present in the B strain (Figure S4 in Additional file 1). It is generally assumed that laboratory strains of *E. coli *are unable to secrete many proteins because the operon encoding the T2S pathway (*gsp*) is silenced by the H-NS protein [31]. The expression levels of *gspB-O *genes, except for *gspE*, were relatively low in both *E. coli *B and K-12 strains throughout the culture period. Expression of the additional T2S genes present in the B strain was detected by transcriptome analysis. Regarding secretion capacity, both REL606 and BL21(DE3) secreted larger amounts of protein than the K-12 strains did (Figure 3). All *E. coli *strains tested secreted greater amounts of protein when grown in minimal media, and the release of proteins was optimal in the stationary phase. Thus, the existence of the second T2S secretion system and enhanced capability for protein release suggest that B strains might be better suited for extracellular production of recombinant proteins.

### *In silico *complementation test revealed genetic bases of the observed phenotypes

To identify the reactions responsible for the phenotypes unique to REL606 compared with MG1655, *in silico *complementation experiments were performed by reverting each of the REL606-specific modifications back to MG1655-specific reactions. Each combination of additions, deletions, and regulations unique to REL606 was reverted and growth under each condition where REL606 and MG1655 show differential substrate utilization phenotypes was predicted using the metabolic network models. Combinations of modifications whose reversion resulted in loss of REL606-specific phenotypes were regarded as reactions responsible for phenotypes unique to REL606.

*In silico *complementation experiments of the reconstructed model verified 23 reactions responsible for the phenotypes unique to REL606 (Table 1). The model can be further applied to test hypotheses derived from sequence and expression data; for example, slow growth of K-12 in pyrimidine-free media can be attributed to the frame-shift mutation of *rph*, thereby decreasing the expression of *pyrE *[32]. FBA results demonstrated that the flux level associated with *pyrE *shows linear correlation with the specific growth rate. Indeed, the *rph *gene was found to be intact in B strains, which grow better than K-12 strains in minimal media (Figure S1B in Additional file 1). Reconstruction of a computational metabolic network of *E. coli *B and *in silico *complementation experiments revealed genetic bases behind the phenotypic characteristics of B.

**Table 1:** Different phenotypes of *E. coli *B REL606 and *E. coli *K-12 MG1655 and modifications of the corresponding reactions

	PM^a^	Prediction^b^		
Carbon source	B/K-12	B/K-12	Responsible reaction^c^	Modification
L-Arabinose	-/++	-/+	ARAI	Deletion
Succinic acid	+/++	-/+	EX_succ(e)	Turned off
L-Aspartic acid	+/++	-/+	EX_asp_L(e)	Turned off
L-Fucose	-/++	-/+	FUCtpp	Deletion
D-Malic acid	-/++	-/+	EX_mal_D(e), MALDt2_2pp	Turned off
L-Malic acid	+/++	-/+	EX_mal_L(e)	Turned off
p-Hydroxyphenyl acetic acid	++/-	+/-	4H3M, 34DH23OR, 5C2HMSO, 5C2HMDI, 5O3E125TC, HPAI, HPAP, HPAtex, EX_4hpa(e)	Addition
N-Acetyl-D-galactosamine	++/-	+/-	ACGALptspp, ACGAL6PI	Addition
D-Arabinose	++/-	+/-	ARABDI, RBK_D1, DARBtex, EX_arab-D(e)	Addition
N-Acetyl-L-glutamic acid	++/-	+/-	NACGLUtmp	Addition

The different phenotypes of *E. coli *B REL606 and *E. coli *K-12 MG1655 were determined from PM tests and modification of the corresponding reactions were verified by *in silico *complementation tests. Experimental and *in silico *cell growth on carbon sources are fully listed in Table S5 in Additional file 1. ^a^The experimental phenotypes are classified into no growth ('-'), low growth ('+'), or high growth ('++') based on growth area. See footnotes of Table S5 in Additional file 1 for details. ^b^*In silico *prediction of cell growth on each carbon source was performed by flux balance analysis: no growth ('-'), growth ('+'). ^c^The metabolic reactions and their associated genes are provided in Table S4 in Additional file 1.

## Conclusions

Despite advances in various high-throughput technologies, extraction of discernable biological meaning from multiple omics data sets remains a challenging task [9,10,13,33]. It has been emphasized that integration of multiple omics results is important and work often focuses on its usage in constructing and validating '*in silico *models'. But how the different and nonlinearly correlated omics data can be combined in the real world is hardly commented on. In this study, we identified many important differences in cellular metabolism and physiology between *E. coli *B and K-12 strains through a combined and integrated analysis of genomic, transcriptomic, proteomic, and phenomic data (Figure 5). Differences in utilization of a variety of nutrient substrates were identified and in many cases could be correlated with genotypic and functional differences. Moreover, transcriptome and proteome data cross-validated information on differentially expressed genes and proteins, demonstrating the utility of integrating omics data collected at various levels from the genome to phenome. Genome-scale *in silico *models can generate and validate hypotheses concerning cellular processes.

Derivatives of *E. coli *B have been the major workhorse for production of recombinant foreign proteins and various biomaterials, including biofuels in the labs and in industry [1,2]. Until now, the bioprocess optimization using B has been carried out in a rather trial-and-error manner. This inefficient approach has been unavoidable because of insufficient information about the metabolism and physiology of B. Therefore, the combined omics approach applied here to the cellular metabolism and physiology of *E. coli *B and K-12 strains should be pivotal in better understanding the underlying biological networks, and invaluable for designing strains with customized genomes and establishing rational fermentation strategies. Moreover, this omics-based systems approach will provide a framework for elucidating the phenetic characteristics of other organisms whose genomes are sequenced.

## Materials and methods

### Strains and culture conditions

*E. coli *B REL606 (F^- ^*mal^+ ^*l*^s ^T6^r ^str^r ^rm*_111 _*ara^-^*) was provided by Richard E Lenski, Michigan State University, and *E. coli *BL21(DE3) was obtained from F William Studier, Brookhaven National Laboratory. *E. coli *K-12 MG1655 was purchased from American Type Culture Collection (ATCC) and *E. coli *W3110 from the Korean Collection for Type Cultures (KCTC). Cells were grown in 250-ml flasks containing 100 ml LB medium or minimal R/2 medium (pH 6.8) supplemented with 10 g/L glucose in a shaking incubator at 37°C and 200 rpm. R/2 medium contains 2 g/L (NH_4_)_2_HPO_4_, 6.75 g/L KH_2_PO_4_, 0.85 g/L citric acid, 0.7 g/L MgSO_4_·7H_2_O, and 5 ml/L of a trace metal solution [34]. Trace metal solution contains 5 M HCl, 10 g/L FeSO_4_·7H_2_O, 2.25 g/L ZnSO_4_·7H_2_O, 1 g/L CuSO_4_·5H_2_O, 0.5 g/L MnSO_4_·5H_2_O, 0.23 g/L Na_2_B_4_O_7_·10H_2_O, 2 g/L CaCl_2_·2H_2_O, and 0.1 g/L (NH_4_)_6_MO_7_O_24_.

### Analytical procedures

Cell growth was monitored by measuring absorbance at 600 nm using an Ultrospec 3000 spectrophotometer (Amersham Biosciences, Uppsala, Sweden). The concentration of glucose and organic acids was determined by high-performance liquid chromatography (ProStar 210, Agilent Technologies, Santa Clara, CA, USA) with UV/visible-light (ProStar 320) and refractive index detectors (Shodex RI-71, Yokohama, Japan). A MetaCarb 87H column (300 × 7.8 mm; Varian) was eluted isocratically with 0.01 M H_2_SO_4 _at 60°C at a flow rate of 0.6 ml/minute.

### Genome analysis

Detailed procedures for genome sequencing and annotation of REL606 have been described previously [6]. RefSeq genome data for MG1655 as of 7 June 2006 [GenBank:NC_000913] were used for comparative genomic analysis. Alignments were generated by the MUMmer 3.19 package. Genomic segments specific to each genome were identified by selecting regions >2 kb that were not aligned to each other at the equivalent position. Pseudogenes in each genome caused by IS insertion, frameshift, or in-frame stop codons were checked using the homologous gene from the other genome by BLAST or CROSS_MATCH. Genomic islands in REL606 and MG1655 were identified as described previously [35]. Briefly, a gene was considered foreign if its G+C content (>1.5 σ) and codon usage (*P *< 0.05) were both aberrant. A genome scan was then performed using a ten-gene window, and regions containing four or more foreign genes were identified as genomic islands, with neighboring islands merged into larger ones.

### Construction of oligonucleotide microarray

Oligonucleotide microarrays were constructed in which both REL606 and MG1655 could be tested for the synthesis of both common and unique mRNAs. Probes (70-mers) were designed by Array Designer (Premier Biosoft International, Palo Alto, CA, USA) and cross-hybridization was checked by BLASTN. A total of 4,819 probes were synthesized by Illumina. The probes represented 3,762 genes that shared over 80 nucleotides of sequence between the two *E. coli *strains, 382 genes found uniquely in REL606, and 534 genes unique to MG1655. The remaining probes were positive and negative controls. The probes were spotted in duplicate onto a GAPSII glass slide (Corning Inc., Corning, NY, USA) by Digital Genomics in Seoul, Korea.

### Transcriptome analysis

The transcriptome of REL606 cells at the exponential growth stage was compared with that of MG1655 cells at the same stage, and with the transcriptome of both REL606 and MG1655 cells in stationary phase. Total RNA was isolated from cells grown in LB medium using the Qiagen RNeasy column (Valencia, CA, USA). Fluorescently labeled cDNA was synthesized by reverse transcription of total RNA with aminoallyl-dUTP and random primers followed by the coupling of Cy3 or Cy5 dyes (Amersham Pharmacia, Uppsala, Sweden). Hybridization and washes were performed according to the manufacturer's instructions (Digital Genomics, Seoul, Korea). The hybridized slides were scanned with GenePix 4000B (Molecular Devices, Sunnyvale, CA, USA) and the scanned images were analyzed by GenePix Pro 6.0. Following background subtraction, spot signal intensities were calculated as percentages of total signal intensities of Cy3 or Cy5 signals on each microarray as a means of normalization. Spots with low intensities were excluded. All microarray experiments were performed in duplicate with dye-swapping. The log_2 _of the intensity ratio with the two dyes for each spot was calculated from up to four values from the duplicated spot on the microarray and the dye-swap experiment. The microarray experiments were highly reproducible: the correlation coefficients between the same spots in each microarray experiment were about 0.98 and correlations between dye-swap paired microarrays were over 0.91. Genes showing growth stage-specific differences in expression levels of ≥2- or ≤0.5-fold were considered to be differentially expressed genes. The microarray data have been deposited at the Gene Expression Omnibus (GEO) [36] under accession number GSE13011.

### Proteome analysis

Whole cellular, outer membrane, and extracellular proteins were extracted and analyzed by two-dimensional electrophoresis using the IPGphor isoelectric focusing system (GE Healthcare (Waukesha, WI, USA) and a Protean II xi electrophoresis cell (Bio-Rad, Hercules, CA, USA) as described previously [37] (see detailed procedures in Supplementary methods in Additional file 1). For protein identification, samples were prepared as described previously [38]. Briefly, gel pieces were proteolysed with 0.02 μg/μl modified trypsin (Promega, Madison, WI, USA) in 40 mM ammonium bicarbonate overnight at 37°C. Tryptic peptides (10 μl aliquots) were analyzed with a nano-liquid chromatography/mass spectrometry system consisting of an Ultimate HPLC system (LC Packings, Sunnyvale, CA, USA) and a quadrupole-time-of-flight mass spectrometry (Micromass, Milford, MA, USA) equipped with a nano-electrospray ionization source, as described previously [37]. The resulting tryptic peptide fragment data were analyzed using the MASCOT search server [39] for identification of protein spots. Reference databases for the identification of target proteins were an in-house *E. coli *B database, UniProt Knowledgebase [40], and NCBI [41].

### Phenome analysis

PM tests on REL606 and MG1655 strains were carried out essentially as described previously [42]. The PM plates (Biolog Inc., Hayward, CA, USA) consisted of 20 96-well microplates containing different sources of carbon (PM1 and PM2), nitrogen (PM3), phosphorus and sulfur (PM4), auxotrophic supplements (PM5 to PM8), or salt (PM9). The PM10 plate tests a pH stress, and plates PM11 to PM20 contain inhibitory compounds such as antibiotics, antimetabolites, and other inhibitors. Cells were grown on BUG+B agar overnight at 37°C. Colonies were picked from the agar surface and suspended in inoculating fluid containing the indicator dye tetrazolium violet. IF-0 inoculating fluid was used for plates PM1 to PM8 and IF-10 fluid for plates PM9 to PM20. Disodium succinate and ferric citrate were added to the inoculation solutions of plates PM3 to PM8. All PM plates were inoculated with cell suspensions at 100 μl/well and incubated at 37°C for 48 h in an OmniLog incubator (Biolog Inc.). For each well, the unitless areas beneath the growth-time curves for REL606 and MG1655 were determined by OmniLog-PM software and were averaged for four PM tests. These values were then subtracted from that of the negative control. Four independent tests were performed on each strain.

### Metabolic network reconstruction and flux balance analysis

Based on the metabolic network model of MG1655 (iAF1260) and genome annotation of REL606 [6], reactions that depend on MG1655-specific genes and pseudogenes were excluded from the metabolic network model for REL606. In addition, reactions that should be turned off by REL606-specific regulation were determined using the regulatory network model for MG1655 constructed by Covert *et al*. [13]. Reactions unique to REL606 were identified by genome annotation and added to the metabolic network model for it.

FBA [43] was used to predict cell growth on various substrates using the reconstructed metabolic network for REL606 and iAF1260 for MG1655. The predicted cell growth was compared with the results from the PM tests [13]. Pre-specified uptake rates of each carbon source (8 mmol/gDCW/h) and oxygen (18.5 mmol/gDCW/h) were used for prediction of cell growth on PM plates testing carbon source utilization (PM1 and PM2). To predict cell growth on PM plates testing utilization of nitrogen (PM3), phosphate (PM4), and sulfur (PM4) sources, the uptake rate of each source (8 mmol/gDCW/h), succinate uptake rate (8 mmol/gDCW/h), and oxygen uptake rate (18.5 mmol/gDCW/h) were prespecified for FBA using the metabolic network models of REL606 and MG1655. The biomass objective function core [15] was used as an objective function. Cell growth on various media conditions was determined by the GNU linear programming kit [44].

## Abbreviations

FBA: flux balance analysis; COG: Clusters of Orthologous Group of proteins; IS: insertion sequence; LB: Luria-Bertani; LPS: lipopolysaccharide; PM: phenotype microarray;T2S: type II secretion; TA: toxin-antitoxin; TCA: tricarboxylic acid.

## Competing interests

The authors declare that they have no competing interests.

## Authors' contributions

JFK conceived, organized and supervised the project, and analyzed the omics information; SHY designed DNA microarray and phenotype microarray experiments, and analyzed and accessed the biological significance of genome, transcriptome, proteome, and phenome data; M-JH and X-XX designed and performed proteome experiments, and analyzed the data; HJ contributed to analyzing the genomic differences between B and K-12; CHL reconstructed the metabolic model of B and performed *in silico *complementation tests; JHS performed the phylogenetic analysis of type II secretion systems; SYL and TKO participated in conceiving the project and analyzing the biological implications of omics data; SHY and M-JH wrote the manuscript; and JFK, SYL, and D-HL revised the draft. All authors have read and approved the manuscript for publication.

## Supplementary Material

Additional file 1 **Supplementary Methods, Figures S1 to S4, Tables S1 to S5, and Supplementary References**. Supplementary Methods: preparation of whole cellular, membrane and extracellular proteins, two-dimensional gel electrophoresis and image analysis, and metabolite abbreviations in Figure 4. Figure S1: growth curves of *E. coli *B strains (REL606 and BL21(DE3) and K-12 strains (MG1655 and W3110) in complex LB medium and minimal R/2 medium supplemented with 10 g/l glucose. Figure S2: total cellular, outer membrane, and extracellular proteomes of the *E. coli *strains. Figure S3: phenotype microarray comparison of *E. coli *B REL606 and K-12 MG1655. Figure S4: phylogenetic position of two T2S systems in *E. coli *B REL606. Table S1: pseudogene comparison between *E. coli *B REL606 and K-12 MG1655. Table S2: genes that were highly expressed at both the exponential and stationary growth phases during growth of *E. coli *B REL606 and K-12 MG1655 in LB medium. Table S3: proteins exhibiting significant quantitative differences between *E. coli *B and K-12 strains. Table S4: metabolic reactions modified in the metabolic network model for *E. coli *B REL606 compared to the model for *E. coli *K-12 MG1655. Table S5: phenotypic differences of *E. coli *B REL606 and K-12 MG1655 in PM1 and PM2 and *in silico *predictions of cell growth on each carbon source. Supplementary References. Click here for file
