# Supplementary material for: Comparative multi-omics systems analysis of Escherichia coli strains B and K-12
Source: Genome Biol. 2012 May 25;13(5):R37. doi: 10.1186/gb-2012-13-5-r37 (PMC3446290; doi:10.1186/gb-2012-13-5-r37)
Supplement: Additional file 1 — Supplementary Methods, Figures S1 to S4, Tables S1 to S5, and Supplementary References. Supplementary Methods: preparation of whole cellular, membrane and extracellular proteins, two-dimensional gel electrophoresis and image analysis, and metabolite abbreviations in Figure 4. Figure S1: growth curves of E. coli B strains (REL606 and BL21(DE3) and K-12 strains (MG1655 and W3110) in complex LB medium and minimal R/2 medium supplemented with 10 g/l glucose. Figure S2: total cellular, outer membrane, and extracellular proteomes of the E. coli strains. Figure S3: phenotype microarray comparison of E. coli B REL606 and K-12 MG1655. Figure S4: phylogenetic position of two T2S systems in E. coli B REL606. Table S1: pseudogene comparison between E. coli B REL606 and K-12 MG1655. Table S2: genes that were highly expressed at both the exponential and stationary growth phases during growth of E. coli B REL606 and K-12 MG1655 in LB medium. Table S3: proteins exhibiting significant quantitative differences between E. coli B and K-12 strains. Table S4: metabolic reactions modified in the metabolic network model for E. coli B REL606 compared to the model for E. coli K-12 MG1655. Table S5: phenotypic differences of E. coli B REL606 and K-12 MG1655 in PM1 and PM2 and in silico predictions of cell growth on each carbon source. Supplementary References. [file gb-2012-13-5-r37-S1.PDF]

ADDITIONAL MATERIAL

Supplementary Methods

Preparation of whole cellular, membrane and extracellular proteins

To extract whole cellular proteins, cells were harvested by centrifugation for 5 min at 3500 × g and 4 °C, and washed four times with low-salt washing buffer (3 mM KCl, 1.5 mM KH_2PO_4, 68 mM NaCl, and 9 mM NaH_2PO_4). The pellet was then resuspended in 600 µl of a buffer containing 10 mM Tris-HCl (pH 8.0), 1.5 mM $MgCl_2$, 10 mM KCl, 0.5 mM DTT, and 0.1% SDS. One µl of this sample was mixed with 60 µl of a solution consisting of 8 M urea, 4% (w/v) CHAPS, 40 mM Tris, 65 mM DTT, and a trace of bromophenol blue, centrifugated at 12,000 × g for 15 min at 15 °C, and collected the supernatant containg whole cellular proteins.

For fractionation of outer membrane proteins, culture broth (3 ml) was centrifuged at 3500 × g for 5 min at 4 °C, and the pellet was washed with 1 ml of 10 mM Na_2HPO_4 buffer (pH 7.2), followed by centrifugation at 3500 × g for 5 min at 4 °C. The cell pellet was resuspended in 0.5 ml of 10 mM Na_2HPO_4 buffer (pH 7.2). Crude extracts of *E. coli* cells were prepared by three cycles of sonication (each for 20 s at 15% of maximum output; high-intensity ultrasonic liquid processors; Sonics & Material Inc.). Partially disrupted cells were first removed by centrifugation of sonicated samples at 12,000 × g for 2 min at room temperature. Membrane proteins and lipid layers were isolated by centrifugation at 12,000 × g for 30 min at 4 °C, followed by resuspension in 0.5 ml of 0.5% (w/v) sarcosyl in 10 mM Na_2HPO_4 buffer (pH 7.2). After incubation at 37 °C for 30 min, the insoluble pellet containing membrane proteins was obtained by centrifugation at 12,000 × g for 30 min at 4 °C. Membrane proteins were obtained by washing the insoluble pellet with 10 mM Na_2HPO_4 buffer (pH 7.2) followed by resuspending in 50 µl of Tris-EDTA buffer (pH 8.0).

For preparation of extracellular proteins, cells were removed by centrifugation at 3500 × g for 5 min at 4 °C. The clear supernatant was collected, passed through a 0.22 µm filter, mixed with equal volume of cold 20% (w/v) TCA (trichloroacetic acid; Sigma) in acetone and kept at -20 °C for 1 h. The precipitate was collected by centrifugation at 13,000 × g for 30 min at 4 °C. The precipitated protein was further washed with acetone to remove traces of TCA, and acetone was finally removed by speed vacuum treatment. The protein pellets were resolubilized in sample rehydration buffer (8 M urea, 2% (w/v) CHAPS, 20 mM DTT, 1% (v/v) cocktail protease inhibitor (Complete Mini EDTA-free; Roche Diagnostics GmbH) and 0.8% (v/v) IPG buffer (Amersham Biosciences). Insoluble materials were removed by centrifugation at 13,000 × g for 10 min. The supernatant was collected, and total soluble protein concentration was measured using the Bradford method (Bradford, 1976). The resulting solution was stored at -80 °C for 2-DE analysis. Total nucleic acids in culture supernatants were measured fluorimetrically using the fluorescent dye bisBenzimide (Sigma; St. Louis, MO) (Moe et al, 1994). No detectable nucleic acids were presented in the culture supernatant, indicating that extracellular protein accumulation is not caused by simple cell lysis, but by a mechanism causing specific release of selected host-cell proteins into the environment.

2-DE and image analysis

Proteins (100 or 200 µg) were diluted with sample rehydration buffer and carefully loaded onto the IPG strips (18-cm, pH 3-10 NL; Amersham Biosciences). The loaded IPG strips were rehydrated for 12 h and focused at 20 °C for 15 min at 250 V, followed by 8,000 V until a total of 60 kV·h was reached. The strips were equilibrated for 15 min in the equilibration buffer I (6 M urea, 0.375 M Tris-HCl (pH 8.8), 20% (w/v) glycerol, 2% (w/v) SDS, and 130 mM DTT), followed by incubation in the equilibration buffer II (6 M urea, 0.375 M Tris-HCl (pH 8.8), 20% (w/v) glycerol, 2% (w/v) SDS, and 135 mM iodoacetamide) for 15 min, and then placed on 12% (w/v) SDS-PAGE gels as described by Laemmli (Laemmli, 1970). Proteins spots were visualized using a silver staining kit (Amersham Biosciences) and the stained gels

were scanned by a UMAX PowerLook 2100XL Scanner (UMAX Technologies, Inc.). PDQuest 2-D Analysis Software (Bio-Rad) was used to automate the process of finding protein spots within the image. To check the reproducibility, 2-DE experiment was carried out in triplicate for independent samples taken from flask cultures.

Metabolite abbreviations in Figure 4.

Intracellular metabolites

13PDG	1,3-bis-Phosphoglycerate
3PG	3-Phosphoglycerate
ABUT	2-Aceto-2-hydroxy butyrate
AC	Acetate
ACCOA	Acetyl-CoA
ACLAC	Acetolactate
AKG	α-Ketoglutarate
ALA	Alanine
ARG	Arginine
ASER	O-Acetylserine
ASN	Asparagine
ASP	Aspartate
ASPSA	Aspartic beta-semialdehyde
CHOR	Chorisimate
CIT	Citrate
CYS	Cysteine
F6P	Fructose 6-phosphate
FDP	Fructose 1,6-diphosphate
FUM	Fumarate
G6P	Glucose 6-phosphate
GLN	Glutamine
GLU	Glutamate
GLY	Glycine
HIS	Histidine
HSER	Homoserine
ICIT	Isocitrate
ILE	Isoleucine
LEU	Leucine
LYS	L-Lysine
MAL	Malate
MET	Methionine
OA	Oxaloacetate
OBUT	Oxobutyrate
OIVAL	Oxoisovalerate
PEP	Phosphoenolpyruvate
PHE	Phenylalanine
PRO	Proline
PYR	Pyruvate
R5P	Ribose 5-phosphate
RL5P	Ribulose 5-phosphate
SER	Serine
SUCC	Succinate
SUCCOA	Succinate CoA
T3P1	Glyceraldehyde 3-phosphate
T3P2	Dihydroxyacetone phosphate
THR	Threonine
TRP	Tryptophan
TYR	Tyrosine
VAL	Valine

Substrates in PM wells

AHBA	α-Hydroxy butyric acid
AKBA	α-Keto-butyric acid
AKG	α-Keto-glutaric acid
BSUCC	Bromo succinic acid
D,L-MAL	D,L-Malic acid
D-ALL	β-D-Allose
D-ARA	D-Arabinose
D-FRU	D-Fructose
D-GLC	α-D-Glucose
D-MAL	D-Malic acid
D-XYL	D-Xylose
FUM	Fumaric acid
GALNAC	N-Acetyl-D-galactosamine
GLUNAC	N-Acetyl-L-glutamic acid
L-ARA	L-Arabinose
L-ASP	L-Aspartic acid
L-ASN	L-Asparagine
L-FUC	L-Fucose
L-GAGL	L-Galactonic acid-g-lactone
L-GLN	L-Glutamine
L-GLU	L-Glutamic acid
L-MAL	L-Malic acid
L-PRO	L-Proline
L-THR	L-Threonine
MEL	Melibionic acid
m-TAR	m-Tartaric acid
p-HPA	p-Hydroxy phenyl acetic acid
SUCC	Succinic acid

Supplementary Figures

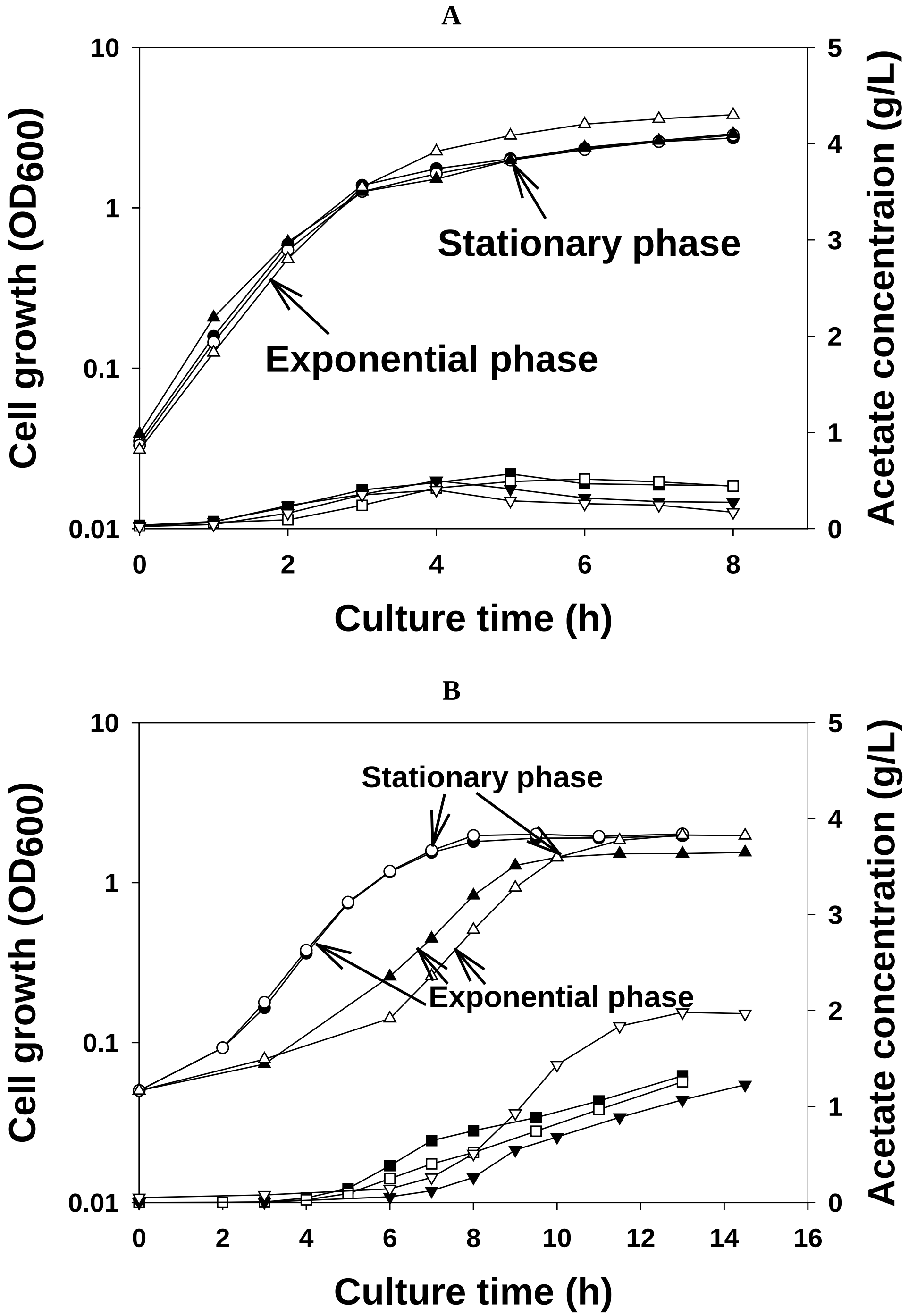

Figure S1. Growth curves of *E. coli* B strains (REL606 (●) and BL21(DE3) (○)) and K-12 strains (MG1655 (▲) and W3110 (∆)) in complex LB medium (**A**) and minimal R/2 medium supplemented with 10 g/L glucose (**B**). Bacterial cells cultured in LB medium were harvested at exponential and stationary phases for transcriptome and proteome analyses. Triplicate growth cultures were tested. Acetate accumulation in the culture broth is shown (REL606 (■), BL21(DE3) (□), MG1655 (▼) and W3110 (▽)). Accumulation of succinic, formic, pyruvic, and lactic acid was similar between B and K-12 strains (data not shown).

A

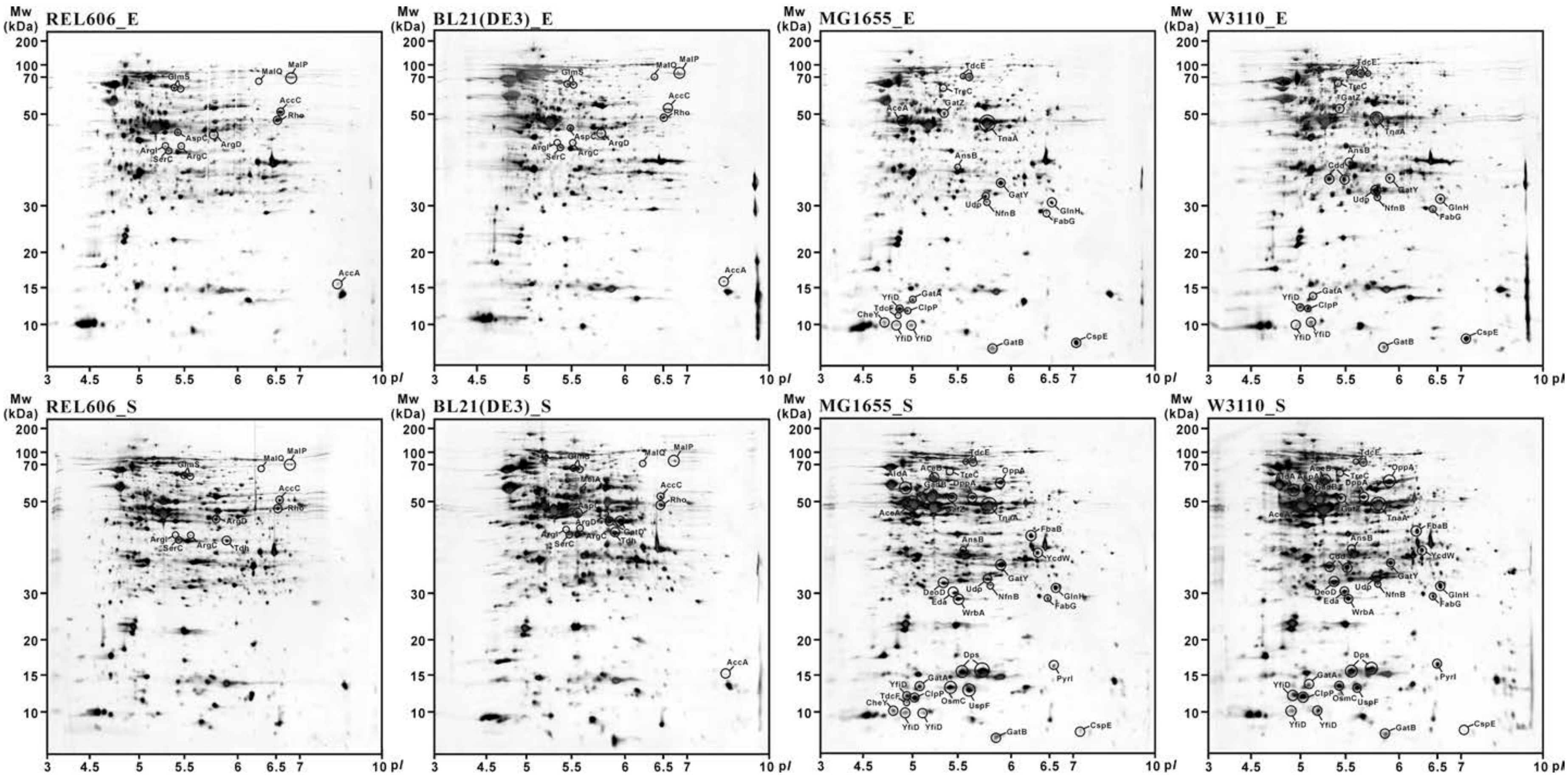

Figure S2. Total cellular, outer membrane, and extracellular proteomes of the *E. coli* strains. (**A**) 2-D gels of whole cellular proteins of *E. coli* B strains (REL606 and BL21(DE3)) and K-12 strains (MG1655 and W3110) during the exponential (upper panels) and stationary phase (lower panels) in LB medium. Proteins that show significantly different levels are indicated by circles on each 2-D gel. (**B**) SDS-PAGE of outer membrane proteins of *E. coli* B and K-12 strains cultured in LB medium. Lane M, protein marker; lane 1, BL21(DE3); lane 2, W3110; lane 3, REL606; lane 4, MG1655. Well-separated OMPs are shown in the lower panel. (**C**) 2-D gels of extracellular proteins of *E. coli* strains at the stationary phase. Extracellular proteins were precipitated from the supernatant of *E. coli* cells grown in defined medium flask cultures. The positions of identified proteins are indicated.

B

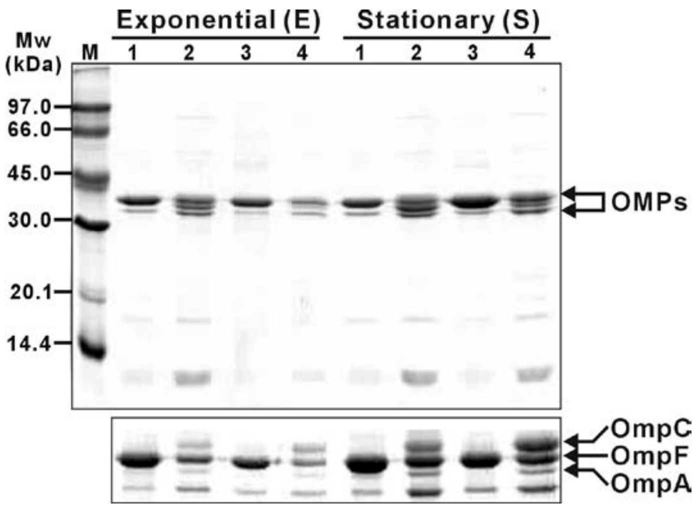
Exponential (E)
Stationary (S)
Mw (kDa)
M
1
2
3
4
97.0
66.0
45.0
30.0
20.1
14.4
OMPs
OmpC
OmpF
OmpA

C

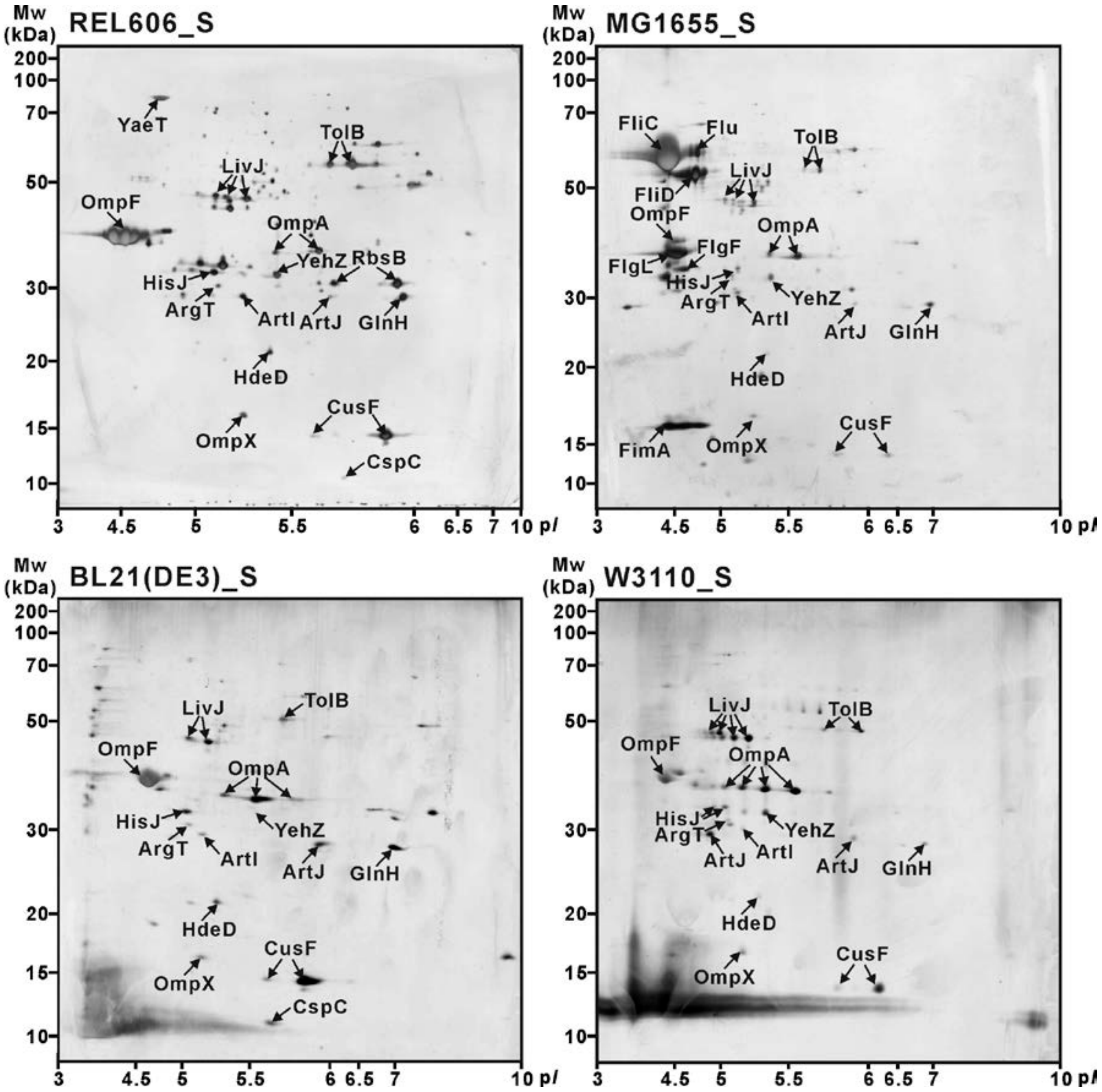
REL606_S
MG1655_S
BL21(DE3)_S
W3110_S
Mw (kDa)
200
100
70
50
30
20
15
10
3
4.5
5
5.5
6
6.5
7
10 pI
YaeT
TolB
LivJ
OmpF
OmpA
YehZ
RbsB
HisJ
ArgT
ArtI
ArtJ
GlnH
HdeD
CusF
OmpX
CspC
FliC
Flu
FliD
FlgF
FlgL
FimA

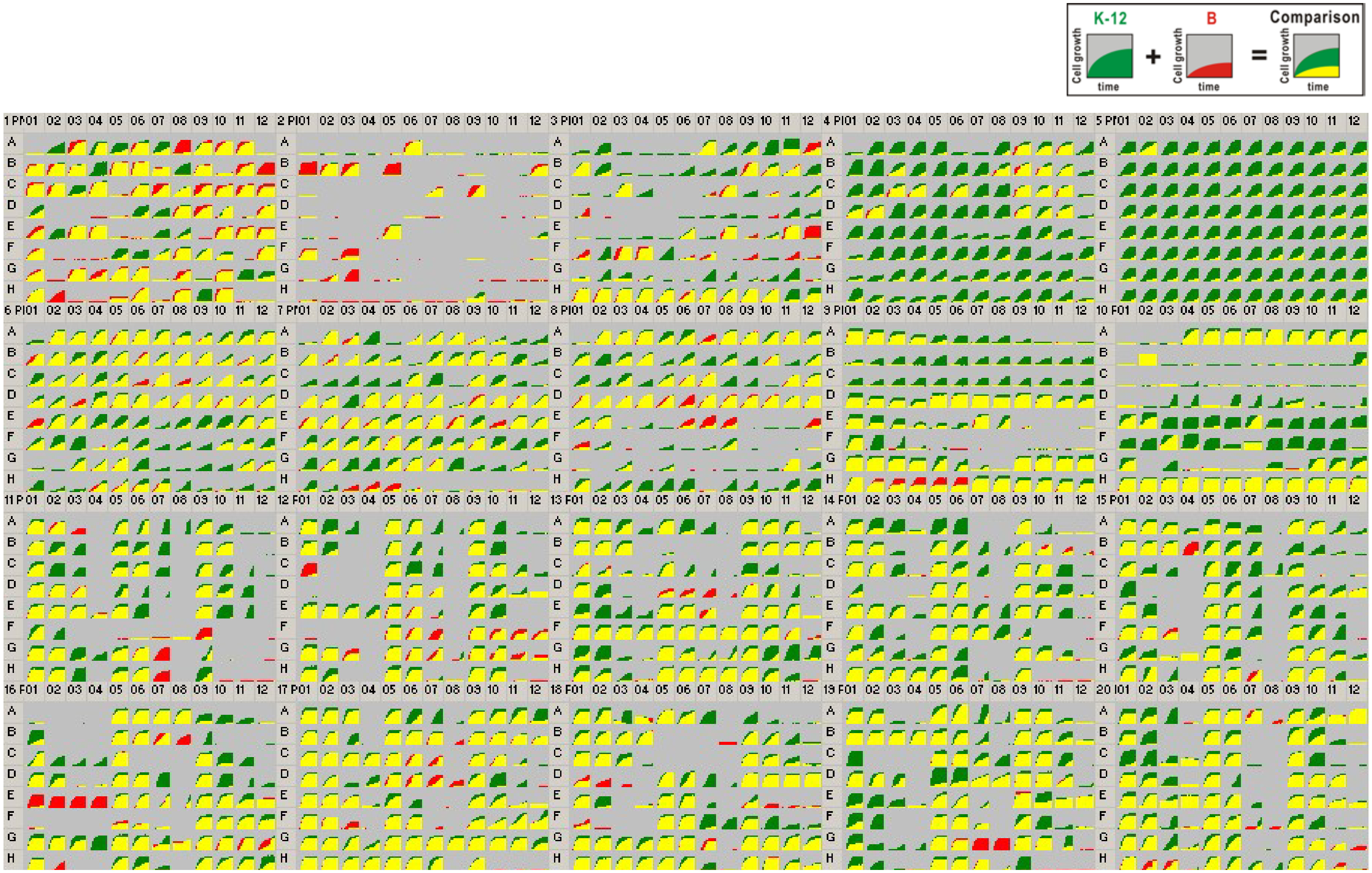

Figure S3. Phenotype microarray comparison of *E. coli* B REL606 and K-12 MG1655. Growth curves are colored yellow for similar growth of REL606 and MG1655, green for faster growth of REL606, and red for faster growth of MG1655. The PM plates (Biolog Inc.) consist of twenty 96-well microplates containing different sources of carbon (PM1 and PM2), nitrogen (PM3), phosphorus and sulphur (PM4), auxotrophic supplement (PM5 to PM8), salt (PM9), pH stress (PM10), and inhibitory compounds such as antibiotics, antimetabolites, and other inhibitors (PM11 to PM20). REL606 displayed severe growth defects on succinic acid as a carbon source. Since disodium succinate was supplemented as the main carbon source (PM3 to PM8), REL606 manifested growth defects in many wells of the PM plates. Detailed information on PM tests can be found at www.biolog.com.

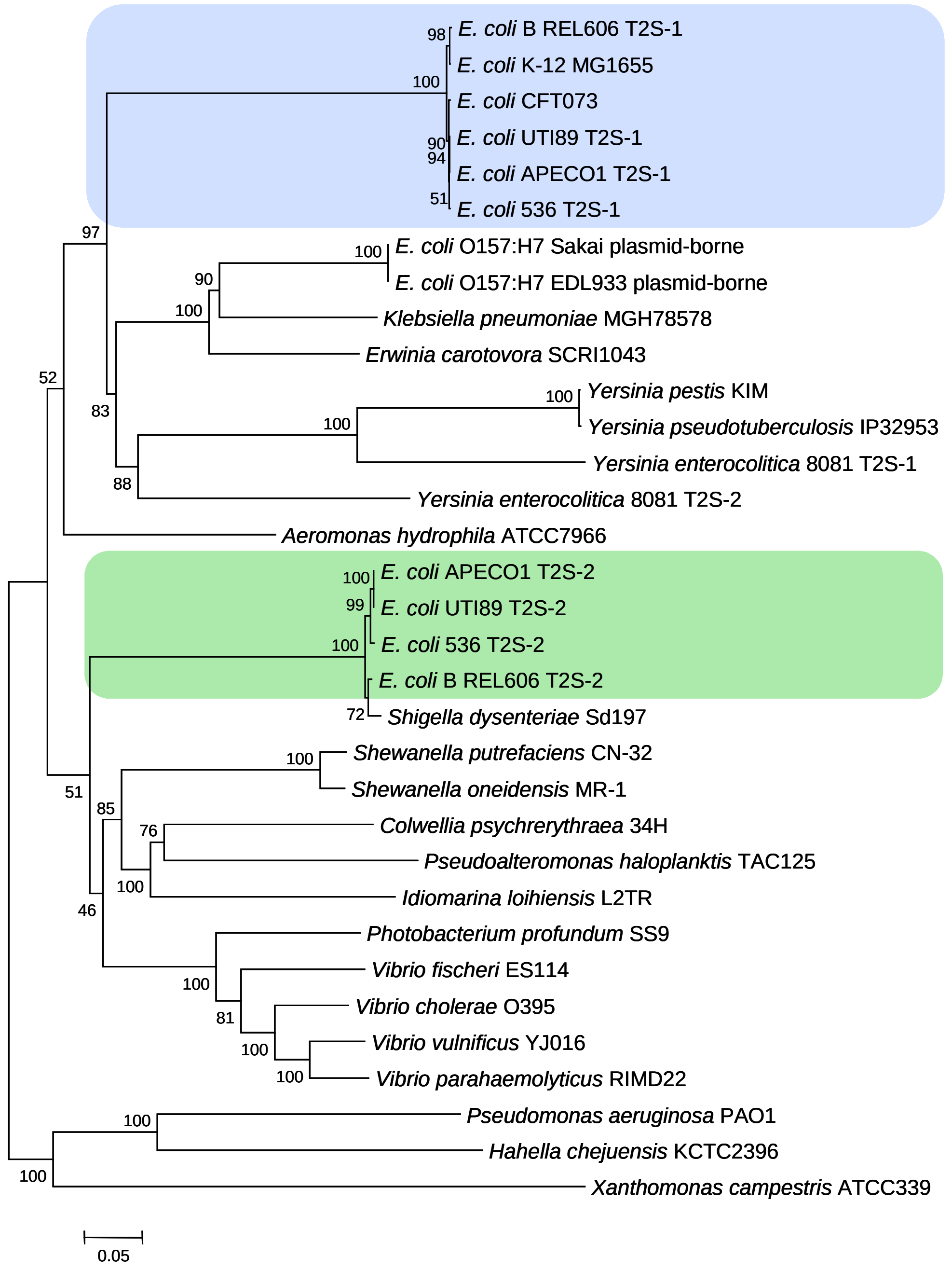

Figure S4. Phylogenetic position of two type II secretion (T2S) systems in *E. coli* B REL606. The phylogenetic tree was inferred from concatenated alignments of four proteins in the T2S system encoding outer membrane secretin (*gspD*), cytoplasmic ATPase (*gspE*), transmembrane protein (*gspF*), and major pseudopilin (*gspG*). Each gene from two T2S systems of REL606 was used as the query in BLASTP searches against the set of ORFs from each of the sequenced genomes of γ-proteobacteria. Multiple alignment of the concatenated sequences was performed by Clustal W, and was subject to the neighbor-joining method to draw a phylogenetic tree by MEGA 4.1 (http://www.megasoftware.net/). In the tree, the common T2S homologs of *E. coli* and other genera in the Enterobacteriaceae formed a single clade, whereas the sequence of the additional T2S in B was grouped in the branch of secondary T2S clusters and those in bacteria other than the Enterobacteriaceae.

Supplementary Tables

Table S1. Pseudogene comparison between *E. coli* B REL606 and K-12 MG1655.

Locus tag	b-number	Gene	REL606[a]	MG1655[a]	Note
ECB_02999	-	*agaW*	C	Pa	*agaW-agaA* deleted in MG1655
ECB_03889	b4017	*arpA*	P	C	
ECB_01689	b4494	*arpB*	Pc	P	
ECB_00042	b0039	*caiB*	P	C	
ECB_04276	b4400	*creD*	P	C	
ECB_00565	b0598	*cstA*	P	C	
ECB_04104	b4236	*cybC*	Pc	Pa	
ECB_03996	b4125	*dcuS*	P	C	
ECB_00224	b0229	*fhiA*	Pc	Pa	Promoterless fragment
ECB_04179	b4312	*fimB*	P	C	Interrupted by IS1
ECB_02645	b2800	*fucA*	P	C	Interrupted by IS1
ECB_01371	b4493	*gapC*	C	P	
ECB_02016	b4498	*gatR*	C	P	Interrupted by IS3
ECB_02021	b2095	*gatZ*	P	C	Interrupted by IS1
ECB_03624	b3740	*gidB*	P	C	
ECB_00618	b0652	*gltL*	P	C	Interrupted by IS150
ECB_03565	b3681	*glvG*	C	Pa	
ECB_01157	b1182	*hlyE*	P	C	
ECB_02873	b2997	*hybO*	P	C	
ECB_02375	b2483	*hyfC*	P	C	
-	b4579	*icdC*	A	P	b4519; in cryptic prophage e14
ECB_03646	b4488	*ilvG*	C	P	
ECB_04161	b4576	*insB*	Pc	P	insB-7 (MG1655), insB-25 (REL606)
ECB_01905	b1997	*insC*	P	C	insC-3 (MG1655), insC (REL606)
ECB_01904	b1996	*insD*	C	Pb	insD-7 (MG1655)
ECB_00544	b3558	*insK*	P	A	Interrupted by IS1
-	b4561	*insM*	A	Pc	
ECB_04151	b4285	*insO*	Pc	Pc	ECB_04151 (REL606), insO-2 (MG1655)
-	b1936	*intG*	A	Pa	
ECB_01547	b1579	*intQ*	P	C	
ECB_01509	b4570	*lomR*	C	P	Interrupted by IS5
ECB_00225	b0230	*mbhA*	Pc	P	Promoterless fragment
ECB_02045	b4499	*molR*	C	P	
ECB_00523	b0569	*nfrB*	P	C	
ECB_00503	b0553	*nmpC*	P	Pa	Interrupted by IS1
-	b2854	*pbl*	A	Pa	
ECB_03975	b4103-b4104	*phnE*	C	Pa	
-	b2648	*pinH*	A	Pa	
ECB_03500	b3643	*rph*	C	Pc	Frameshift mutation in MG1655
-	b1157	*stfE*	A	Pa	b1157; in cryptic prophae e14
-	b0561	*tfaD*	A	Pa	b0561; in cryptic prophage e14
-	b2353	*tfaS*	A	Pa	b2353; in cryptic prophage CPS-53
ECB_01207	b1229	*tpr*	P	C	
ECB_03483	-	*waaT*	P	A	Interrupted by IS1
-	b4571	*wbbL*	A	P	Interrupted by IS1 (rfb-50 mutation)
ECB_03419	b3567	*xylG*	P	C	
-	b4503	*yafF*	A	P	
ECB_00321	b4580	*yaiT*	C	P	Interrupted by IS3
-	b4579	*yaiX*	A	P	
ECB_00352	b0404	*yajB*	P	C	
ECB_00491	b4508	*ybcD*	Pc	Pa	

ECB_00595	b4581	*ybeM*	C	P	
ECB_00613	b0644	*ybeQ*	P	C	
-	b0705	*ybfL*	A	Pa	
-	b4514	*ybfQ*	A	P	
ECB_01019	b4490	*ycdN*	C	P	
ECB_01115	b1119	*ycfX*	P	C	Interrupted by IS1
ECB_01145	b4491	*ycgH*	Pc	P	
-	b4573	*ychG*	A	P	
ECB_01242	b1268	*yciQ*	P	C	Interrupted by IS1
ECB_01252	b4574	*yciX*	Pc	P	
ECB_01295	b1318	*ycjV*	C	P	
-	b4492	*ydbA*	A	P	
ECB_01422	b1464	*yddE*	P	C	
ECB_01471	b1509	*ydeU*	Pc	Pb	
ECB_01627	b1657	*ydhP*	P	C	
ECB_01659	b1690	*ydiM*	P	C	
ECB_01720	b1751	*ydjY*	P	C	
-	b4495	*yedN*	A	P	
-	b4496	*yedS*	A	P	
ECB_01892	b4497	*yeeL*	Pc	P	
ECB_02799	b1999	*yeeP*	Pc	Pa	
ECB_01988	b2083	*yegZ*	C	P	
ECB_02154	b4500	*yfaS*	C	P	
ECB_02197	b2271	*yfbL*	P	C	
ECB_02269	b2345	*yfdF*	P	C	Interrupted by IS1
ECB_02450	b2558	*yfhD*	P	C	
ECB_02536	b2680-b2681	*ygaY*	C	Pa	*ygaX-ygaY* fused in REL606
-	b2858	*ygeN*	A	Pc	
ECB_02830	b2969	*yghE*	Pc	Pa	
ECB_02875	b2999-b3000	*yghX, yghY*	C	Pa	*yghX-yghY* fused in REL606
-	b4569	*yhcE*	A	P	
ECB_03226	b3376	*yhfS*	P	C	Interrupted by IS1
ECB_00665	b3484	*yhhI-3*	P	C	REL606 has three additional complete copies of *yhhI*
ECB_03339	b3489-b3490	*yhiK*	C	Pc	*yhiK-yhiL* fused in REL606
ECB_03382	b3534	*yhjQ*	Pc	Pa	
ECB_03567	b3684	*yidP*	P	C	
ECB_03655	b3777	*yifN*	Pc	Pa	
ECB_03773	b3888	*yiiD*	P	C	
ECB_03933	b4061	*yjcC*	P	C	
ECB_04138	b4575	*yjgX*	Pc	P	
ECB_04143	b4281	*yjhD*	Pc	Pa	
ECB_04144	b4282	*yjhE*	Pc	Pa	
ECB_04175	b4308	*yjhR*	P	C	
-	b4562	*yjhW*	A	P	
ECB_04207	b4338-b4339	*yjiP*	C	Pa	*yjiP-yjiQ* fused in REL606
ECB_04210	b4486	*yjiV*	C	Pa	
ECB_04234	b4357	*yjjM*	P	C	
ECB_00469	b4582	*ylbE*	C	P	
-	b1028	*ymdE*	A	Pc	
-	b4525	*ymjB*	A	P	
ECB_01304	b4525	*ymjC*	Pc	P	
ECB_01389	b4578	*yncK*	Pc	P	
ECB_01548	b4534	*ynfP*	Pc	P	
ECB_01903	b4582	*yoeA*	Pc	P	Interrupted by IS2
-	b4543	*ypaA*	A	P	
-	b4545	*ypdJ*	A	P	

-	b2641	*ypjM*	A	Pa	
-	b2657	*yqaC*	A	Pa	
ECB_03333	b4552	*yrhC*	Pc	P	
-	b2657	*ysdC*	A	P	
ECB_03278	b3427	*yzgL*	P	C	Interrupted by ECB_03279 in REL606
ECB_01464	-		P	A	
ECB_01466	-		P	A	
ECB_01526	-		P	A	
ECB_03528	-		P	A	

[a] Genes were classified as follows; **A**, absent from the genome or no ortholog exists; **C**, complete; **P**, declared as pseudo in "pseudo" qualifier of MG1655 GenBank flat file (gbk) or disrupted by either frameshift, in-frame stop codon, or IS insertion in REL606; **Pa**, pseudogene information in the product description of MG1655 gbk while not given "pseudo" qualifier explicitly; **Pb**, described as predicted pseudogene by MG1655 gbk; **Pc**, predicted to be pseudogene by sequence comparison with its ortholog. MG1655 gbk as of Sep-2006 (GI:49175990) was used for the analysis.

Note that neighboring genes (*glvB-glvC*, *ybfG-ybfH*, *yogG-yogH*, and *yfcT-yfcU*) are fused in REL606, but are separately present in MG1655.

Table S2. Genes that were highly expressed both at the exponential and stationary growth phases during growth of *E. coli* B REL606 and K-12 MG1655 in LB medium.

**Highly expressed genes in B REL606**						**Highly expressed genes in K-12 MG1655**					
**Gene (function[a])**	**Fold change[b]**		**Gene (function)**	**Fold change**		**Gene (function)**	**Fold change**		**Gene (function)**	**Fold change**	
	**B_E/ K12_E**	**B_S/ K12_S**		**B_E/ K12_E**	**B_S/ K12_S**		**B_E/ K12_E**	**B_S/ K12_S**		**B_E/ K12_E**	**B_S/ K12_S**
crl (-)	4.7	6.2	*ilvG* (-)	1.6	2.0	*ompC* (CW)	-7.8	-7.5	*fxsA* (FU)	-3.0	-1.3
ompF (CW)	3.4	6.4	*ilvD* (-)	1.8	1.7	*gatY* (CA)	-6.9	-7.6	*ibpA* (PM)	-2.5	-1.7
cybC (-)	2.6	6.9	*aroP* (AA)	1.4	2.1	*gatZ* (CA)	-6.5	-7.5	*groES* (PM)	-2.9	-1.3
gltS (AA)	3.1	4.3	*argE* (AA)	2.1	1.4	*gatC* (CA)	-5.0	-5.0	*fusA* (TL)	-1.2	-2.7
argB (AA)	4.2	1.8	*hisP* (AA)	1.1	2.3	*cspE* (-)	-6.5	-3.2	*evgS* (ST)	-1.5	-2.4
argD (AA)	3.2	2.6	*malQ* (-)	2.2	1.1	*cheW* (CM)	-5.3	-4.3	*cobB* (TR)	-2.3	-1.6
argH (AA)	4.0	1.6	*ilvA* (AA)	2.0	1.4	*cheA* (CM)	-4.8	-4.5	*fes* (II)	-2.0	-1.8
argC (AA)	3.7	1.9	*ppdB* (IT)	1.3	1.9	*lon* (PM)	-5.5	-3.3	*evgA* (ST)	-1.9	-2.0
thiM (CT)	3.5	1.8	*fabF* (LT)	1.1	2.0	*tap* (CM)	-4.2	-3.9	*uxaC* (CA)	-2.8	-1.0
argG (AA)	3.2	1.6	*hokE* (-)	1.2	1.9	*cheY* (-)	-4.6	-3.1	*entF* (SM)	-1.1	-2.6
argI (AA)	3.1	1.6	*rdgC* (-)	1.9	1.1	*glpA* (EP)	-2.6	-4.6	*nrdI* (NT)	-1.3	-2.4
abgA (FU)	1.1	3.5	*tauD* (SM)	1.1	1.8	*fimA* (CM)	-3.0	-4.1	*treB* (CA)	-2.2	-1.2
malS (-)	2.1	2.5	*ilvE* (AA)	1.5	1.3	*dsdX* (CA)	-4.1	-2.8	*maeB* (EP)	-2.3	-1.0
artJ (AA)	2.8	1.8	*hisJ* (AA)	1.2	1.5	*glpT* (CA)	-2.7	-4.2	*moaB* (-)	-1.4	-1.9
sotB (CA)	2.4	2.1	*prfA* (TL)	1.5	1.2	*aceK* (-)	-3.4	-3.1	*flhC* (-)	-1.8	-1.4
hokD (-)	1.8	2.7	*lrhA* (TR)	1.1	1.6	*trg* (CM)	-3.9	-2.4	*hybC* (EP)	-1.5	-1.6
rho (TR)	2.2	2.2	*setB* (-)	1.4	1.3	*tsr* (CM)	-4.0	-2.2	*entC* (CT)	-1.3	-1.8
cusC (CW)	3.0	1.4	*mdoC* (-)	1.3	1.3	*glpB* (AA)	-2.7	-3.5	*clpB* (PM)	-1.8	-1.1
cusB (CW)	2.9	1.4	*pheP* (AA)	1.4	1.1	*glpQ* (EP)	-2.6	-3.6	*tdcB* (AA)	-1.4	-1.5
malZ (-)	2.8	1.4	*ruvB* (RE)	1.3	1.2	*tdcA* (TR)	-3.2	-2.6	*tdcE* (EP)	-1.7	-1.2
ilvM (FU)	1.9	2.3	*proP* (-)	1.1	1.3	*glpC* (EP)	-2.5	-3.3	*ompA* (CW)	-1.4	-1.5
thiD (CT)	2.6	1.3	*tatE* (-)	1.1	1.3	*lldR* (TR)	-4.3	-1.4	*dnaK* (PM)	-1.4	-1.5
bcsZ (-)	1.8	2.1	*emrE* (II)	1.0	1.3	*lldD* (EP)	-4.3	-1.3	*hyaE* (-)	-1.0	-1.7
fkpA (PM)	1.3	2.5	*speA* (AA)	1.2	1.2	*nmpC* (CW)	-3.3	-2.2	*htpG* (PM)	-1.5	-1.2
uppP (-)	1.9	1.9	*cfa* (CW)	1.3	1.0	*hybO* (EP)	-2.2	-3.2	*eutB* (-)	-1.7	-1.0
rhoL (-)	2.4	1.4	*tilS* (CC)	1.1	1.1	*fhuF* (FU)	-2.8	-2.6	*melR* (-)	-1.3	-1.4
sugE (II)	1.9	1.8	*cysI* (II)	1.0	1.2	*glpD* (EP)	-2.2	-3.0	*fdnG* (EP)	-1.0	-1.7
argA (AA)	2.4	1.3				*cheZ* (CM)	-4.1	-1.1	*dcuS* (ST)	-1.3	-1.4
nrdG (PM)	1.9	1.8				*cheR* (CM)	-3.6	-1.4	*sodA* (II)	-1.0	-1.5
gabT (AA)	1.9	1.7				*hybB* (EP)	-2.5	-1.8	*hipB* (TR)	-1.2	-1.0

Genes showing transcription level of ≥ 2 fold (or ≤ 0.5 fold) both at the exponential and stationary phases were considered as highly expressed in MG1655 (or highly expressed in REL606). Function-unknown genes were not denoted for the simplicity.

[a]Functional categories by Clusters of Orthologous Groups (http://www.ncbi.nlm.nih.gov/COG/). Abbreviations: AA, amino acid transport and metabolism; CA, carbohydrate transport and metabolism; CC, cell cycle control, cell division, chromosome partitioning; CM, cell motility; CW, cell wall/membrane/envelope biogenesis; CT, coenzyme transport and metabolism; EP, energy production and conversion; FU, function unknown; II, inorganic ion transport and metabolism; IT, intracellular trafficking, secretion and vesicular transport; LT, lipid transport and metabolism; NT, nucleotide transport and metabolism; PM, posttranslational modification, protein turnover, chaperones; RE, replication, recombination and repair; SM, secondary metabolites biosynthesis, transport and catabolism; ST, signal transduction mechanisms; TR, transcription; TL, translation, ribosomal structure and biogenesis; -, not assigned

[b]Fold change denotes $\log_2$-transformed transcription ratio of B REL606 to K-12 MG1655 at exponential stage (B_E versus K12_E) and stationary growth phase (B_S versus K12_S).

Table S3. Proteins exhibiting significant quantitative differences between *E. coli* B and K-12 strains.

Gene name[a]	Protein name[a]	SWISS Accession No.[a]	Fractionation[b]	p*I* [a]	Mw[a] (Da)	Score[c]	Fold difference[d]	
							REL606_E/MG1655_E (BL21(DE3)_E/W3110_E)	REL606_S/MG1655_S (BL21(DE3)_S/W3110_S)
Proteins present at substantially elevated levels in B strains								
accA	Acetyl-coenzyme A carboxylase carboxyl transferase subunit alpha	P0ABD5	WC	5.76	35.1	558	+100 (+100)	ND (+100)
accC	Biotin carboxylase	P24182	WC	6.65	49.3	519	+2.5 (+2.1)	+1.3 (+3.8)
argC	N-acetyl-gamma-glutamyl-phosphate reductase	P11446	WC	5.58	36.0	357	+100 (+100)	+100 (+100)
argD	Acetylornithine/succinyldiaminopimelate aminotransferase	P18335	WC	5.80	43.6	520	+100 (+100)	+100 (+100)
argI	Ornithine carbamoyltransferase chain I	P04391	WC	5.46	36.8	745	+100 (+100)	+100 (+100)
aspC	Aspartate aminotransferase	P00509	WC	5.54	43.6	1402	+7 (+100)	+1.2 (+6.3)
cspC	Cold shock-like protein	P0A9Y6	EC	6.82	7.3	373	-	+100 (+100)
cusF	Cation efflux system protein	P77214	EC	6.19	9.9	91	-	+5.4 (+2.6)
glmS	L-glutamine:D-fructose-6-phosphate aminotransferase	P17169	WC	5.56	66.8	452	+2.3 (+2.5)	+3.0 (+3.7)
malP	Maltodextrin phosphorylase	P00490	WC	6.93	90.4	1369	+5.0 (+3.3)	+4.6 (+4.2)
malQ	4-alpha-glucanotransferase	P15977	WC	6.14	78.5	1776	+100 (+100)	+100 (+100)
melA	Alpha-galactosidase	P06720	WC	5.52	50.7	269	ND (ND)	ND (+100)
ompF	Outer membrane protein F	P02931	OM	4.64	37.1	746	+3.3 (+5.2)	+2.6 (+3)
rbsB	D-ribose-binding periplasmic protein	P02925	EC	5.99	28.5	772	-	+100 (ND)
rho	Transcription termination factor	P0AG30	WC	6.75	47.0	565	+5.5 (+2.5)	+3.9 (+2.1)
serC	Phosphoserine aminotransferase	P23721	WC	5.37	39.7	491	+100 (+100)	+100 (+100)
tdh	L-threonine 3-dehydrogenase	P07913	WC	5.94	37.2	254	ND (ND)	+2.8 (+5.0)
yaeT	Outer membrane protein assembly factor	P0A940	EC	4.87	88.4	415	-	+100 (ND)
Proteins present at substantially elevated levels in K-12 strains								
aceA	Isocitrate lyase	P0A9G6	WC	5.16	47.5	1737	-10 (-1.2)	-70 (-50)
aceB	Malate synthase A	P08997	WC	5.39	60.3	563	ND (ND)	-50 (-20)
aldA	Aldehyde dehydrogenase A, NAD-linked	P25553	WC	5.07	52.1	624	ND (ND)	-100 (-100)
ansB	L-asparaginase 2	P00805	WC	5.66	34.6	398	-100 (-100)	-100 (-100)
aspA	Aspartate ammonia-lyase	P0AC38	WC	5.19	52.4	241	+1.0 (-1.4)	-1.2 (-2.3)
cdd	Cytidine deaminase	P0ABF6	WC	5.42	31.5	143	-1.5 (-5.3)	-1.2 (-10)
cheY	Chemotaxis protein	P0AE67	WC	4.89	14.0	35	-100 (ND)	-100 (ND)
clpP	ATP-dependent Clp protease proteolytic subunit	P0A6G7	WC	5.55	21.6	203	-2.5 (-3)	-2.2 (-3.9)
cspE	Cold shock protein E	P0A972	WC	8.06	7.3	62	-100 (-100)	-100 (-100)
deoD	Purine nucleoside phosphorylase	P0ABP8	WC	5.42	25.8	385	+1.1 (-1.3)	-2.2 (-5.3)

dppA	Periplasmic dipeptide transport protein	P23847	WC	5.75	57.4	1193	+1.7 (+1.9)	-2.7 (-10)
dps	DNA protection during starvation protein	P0ABT2	WC	5.72	18.6	417	+1.3 (-1.9)	-3.2 (-3.6)
eda	Multifunctional 2-keto-3-deoxygluconate 6-phosphate aldolase, 2-keto-4-hydroxyglutarate aldolase	P0A955	WC	5.57	22.3	80	-1.2 (+1.3)	-2.2 (-2.1)
fabG	3-oxoacyl-[acyl-carrier-protein] reductase	P0AEK2	WC	6.76	25.6	226	-100 (-100)	-100 (-100)
fbaB	Fructose-bisphosphate aldolase class 1	P0A991	WC	6.24	38.0	423	ND (ND)	-100 (-100)
fimA	Major type 1 subunit fimbrin	P04128	EC	4.60	15.8	74	-	-100 (-100)
flgF	Flagellar basal-body rod protein	P75938	EC	4.80	25.9	30	-	-100 (ND)
flgL	Flagellar hook-associated protein 3	P29744	EC	4.63	34.3	322	-	-100 (ND)
fliC	Flagellar filament structural protein	P04949	EC	4.50	51.2	612	-	-100 (ND)
fliD	Flagellar filament capping protein	P24216	EC	4.82	48.3	418	-	-100 (ND)
flu	Antigen 43 (Ag43) phase-variable biofilm formation autotransporter	P39180	EC	5.77	31.9	203	-	-100 (ND)
gadB	Glutamate decarboxylase beta	P69910	WC	5.29	52.7	688	ND (ND)	-100 (-100)
gatA	Galactitol-specific phosphotransferase enzyme IIA component	P69828	WC	5.15	16.9	59	-100 (-100)	-100 (-100)
gatB	Galactitol-specific phosphotransferase enzyme IIB component	P37188	WC	5.84	10.2	133	-100 (-100)	-100 (-100)
gatY	Tagatose-1,6-bisphosphate aldolase	P0C8J6	WC	5.87	30.8	111	-100 (-100)	-100 (-100)
gatZ	Putative tagatose 6-phosphate kinase	P0C8J8	WC	5.50	47.1	433	-100 (-100)	-100 (-100)
glnH	Glutamine-binding periplasmic protein	P0AEQ3	WC	6.87	25.0	372	-3.3 (-2.5)	-8.9 (-10)
nfnB	Oxygen-insensitive NAD(P)H nitroreductase	P38489	WC	5.80	23.9	93	-100 (-100)	-100 (-100)
ompA	Outer membrane protein A	P0A910	OM	5.60	35.2	631	-3.5 (-2.9)	-10 (-2.6)
ompC	Outer membrane protein C	P06996	OM	4.48	38.3	639	-100 (-100)	-100 (-100)
oppA	Periplasmic oligopeptide-binding protein	P23843	OM	5.85	58.4	1399	-1.8 (-1.4)	-2.5 (-5.0)
osmC	Osmotically inducible peroxidase	P0C0L2	WC	5.57	15.0	127	-1.2 (+1.3)	-5.8 (-6.6)
pyrI	Aspartate carbamoyltransferase regulatory chain	P0A7F3	WC	6.84	17.0	213	ND (ND)	-4.5 (-7.0)
tdcE	Keto-acid formate acetyltransferase	P42632	WC	5.48	86.0	895	-6.2 (-9)	-5.5 (-2.1)
tdcF	Protein tdcF	P0AGL2	WC	5.06	14.0	124	-100 (ND)	-100 (ND)
tnaA	Tryptophanase/L-cysteine desulfhydrase, PLP-dependent	P0A853	WC	5.88	52.8	722	-100 (-100)	-31 (-26)
treC	Trehalose-6-phosphate hydrolase	P28904	WC	5.51	63.8	399	-100 (-100)	-100 (-100)
udp	Uridine phosphorylase	P12758	WC	5.81	27.0	143	-100 (-67)	-43 (-100)
uspF	Universal stress protein F	P37903	WC	5.60	16.0	59	-1.5 (-1.7)	-5.5 (-6.8)
wrbA	Flavoprotein wrbA	P0A8G6	WC	5.60	20.7	327	-1.1 (-1.3)	-2.5 (-4.3)
ycdW(ghrA)	Glyoxylate/hydroxypyruvate reductase A	P75913	WC	6.76	36.8	258	ND (ND)	-7.0 (-5.5)
yfiD(gr	Autonomous glycyl radical cofactor	P68066	WC	5.09	14.3	327	-45 (-44)	-22 (-34)

cA)

[a]The gene name, accession number, description of identified proteins are from ExPASy Proteomics Server (http://kr.expasy.org/). The search is performed on the current UniProt Knowledgebase release (Swiss-Prot and TrEMBL). Calculated p*I* and MW are from ExPASy Proteomics Server.

[b]Fractionation is a method of proteins preparation to find their subcellular localization; WC, whole cellular proteins; OM, outer membrane proteins; and EC, extracellular proteins.

[c]The score is from MASCOT search results of Matrix science (http://www.matrixscience.com/). Individual ions scores > 26 indicate identity or extensive homology ($p < 0.05$).

[d]Fold differences were calculated from at least triplicate spots. All proteins are statistically significant with p values of < 0.05. Spots that were only detected in the *E. coli* REL606 or BL21(DE3) were arbitrarily assigned a fold difference of +100 and spots detected only on *E. coli* MG1655 or W3110 were assigned a fold difference of -100. ND indicates no detection on 2-D gels.

Table S4. Metabolic reactions modified in the metabolic network model for *E. coli* B REL606 as compared to the model for *E. coli* K-12 MG1655.

Abbreviation	Metabolic reaction	Gene association	Modification
ARABDI	[c] : arab-D <==> rbl-D	b2802	addition
RBK_D1	[c] : atp + rbl-D --> adp + h + ru5p-D	ECB_02649	addition
DARBabcpp	arab-D[p] + atp[c] + h2o[c] --> adp[c] + arab-D[c] + h[c] + pi[c]	(b1901 and b1900 and b4460)	addition
DARBt2rpp	arab-D[p] + h[p] <==> arab-D[c] + h[c]	b2841	addition
DARBt3ipp	arab-D[c] + h[p] --> arab-D[p] + h[c]	b1528	addition
DARBtex	arab-D[e] <==> arab-D[p]	(b0241 or b0929 or b1377 or b2215)	addition
4H3M	[c]: 4hpa + o2 + nadh + h <==> 34dhpl + nad + h2o	ECB_04222	addition
34DH23OR	[c] : 34dhpl + o2 <==> 2h5cmmsa	ECB_04228	addition
5C2HMSO	[c] : 2h5cmmsa + nad + h2o <==> 5c2hm + nadh + h	ECB_04229	addition
5C2HMDI	[c] : 5c2hm <==> 5c2o3e	ECB_04227	addition
5O3E125TC	[c] : 5c2o3e <==> 2hh24d + co2	ECB_04230	addition
HPAG	[c] : 2hh24d <==> 2o3e	ECB_04230	addition
2OH3E17DH	[c] : 2o3e + h2o <==> 24dhh2ed	ECB_04226	addition
HPAH	[c] : 2hh24d + h2o <==> 24dhh2ed	ECB_04226	addition
HPAI	[c] : 24dhh2ed <==> sucsal + pyr	ECB_04225	addition
HPAP	4hpa[p] + h[p] <==> 4hpa[c] + h[c]	ECB_04224	addition
HPAtex	4hpa[e] <==> 4hpa[p]	(b0241 or b0929 or b1377 or b2215)	addition
ACGALptspp	acgal[p] + pep[c] --> acgal6p[c] + pyr[c]	ECB_02998,ECB_02999,ECB_03000,ECB_03001,ECB_03002	addition
ACGAL6PI	[c] : acgal6p --> tag6p-D	b3141	addition
GLXtmp	glx[e] --> glx[c]		addition
URATEtmp	urate[e] --> urate[c]		addition
NACGLUtmp	acglu[e] --> acglu[c]		addition
DMAN1Ptmp	man1p[e] -> man1p[c]		addition
EX_arab-D(e)	[e] : arab-D <==>		addition
EX_4hpa(e)	[e] : 4hpa <==>		addition
EX_glx(e)	[e] : glx <==>		addition
EX_urate(e)	[e] : urate <==>		addition
EX_man1p(e)	[e] : man1p <==>		addition
EX_acglu(e)	[e] : acglu <==>		addition
AAMYL	[c] : 14glucan --> malthx	b1927	deletion
ACGAMK	[c] : acgam + atp --> acgam6p + adp + h	b1119	deletion

ADNt2rpp	adn[p] + h[p] <==> adn[c] + h[c]	b2406	deletion
ASPabcpp	asp-L[p] + atp[c] + h2o[c] --> adp[c] + asp-L[c] + h[c] + pi[c]	(b0655 and b0654 and b0653 and b0652)	deletion
CYSDDS	[c] : cys-D + h2o --> h2s + nh4 + pyr	b1919	deletion
CYTDt2rpp	cytd[p] + h[p] <==> cytd[c] + h[c]	b2406	deletion
FA100ACPHi	[c] : dcaACP + h2o --> ACP + dca + h	b0404	deletion
FA120ACPHi	[c] : ddcaACP + h2o --> ACP + ddca + h	b0404	deletion
FA140ACPHi	[c] : h2o + myrsACP --> ACP + h + ttdca	b0404	deletion
FA141ACPHi	[c] : h2o + tdeACP --> ACP + h + ttdcea	b0404	deletion
FA160ACPHi	[c] : h2o + palmACP --> ACP + h + hdca	b0404	deletion
FA161ACPHi	[c] : h2o + hdeACP --> ACP + h + hdcea	b0404	deletion
FA80ACPHi	[c] : h2o + ocACP --> ACP + h + octa	b0404	deletion
FUCtpp	fuc-L[p] + h[p] <==> fuc-L[c] + h[c]	b2801	deletion
GLUabcpp	atp[c] + glu-L[p] + h2o[c] --> adp[c] + glu-L[c] + h[c] + pi[c]	(b0655 and b0654 and b0653 and b0652)	deletion
HCYSMT	[c] : amet + hcys-L --> ahcys + h + met-L	b0261	deletion
HCYSMT2	[c] : hcys-L + mmet --> h + (2) met-L	b0261	deletion
INSt2rpp	h[p] + ins[p] <==> h[c] + ins[c]	b2406	deletion
MMETt2pp	h[p] + mmet[p] --> h[c] + mmet[c]	b0260	deletion
O16AP1pp	[p] : (2) o16aund --> h + o16a2und + udcpdp	(b2035 and b2027)	deletion
O16AP2pp	[p] : o16a2und + o16aund --> h + o16a3und + udcpdp	(b2035 and b2027)	deletion
O16AP3pp	[p] : o16a3und + o16aund --> h + o16a4und + udcpdp	(b2035 and b2027)	deletion
O16AT	[c] : accoa + ragund --> aragund + coa	b2033	deletion
O16AUNDtpp	o16aund[c] --> o16aund[p]	b2037	deletion
O16GALFT	[c] : garagund + udpgalfur --> gfgaragund + h + udp	b2034	deletion
O16GLCT1	[c] : aragund + udpg --> garagund + h + udp	b2032	deletion
PACCOAL	[c] : atp + coa + pac --> amp + phaccoa + ppi	b1398	deletion
PUNP7	[c] : pi + xtsn <==> r1p + xan	b2407	deletion
TDPDRE	[c] : dtdp4d6dg --> dtdp4d6dm	b2038	deletion
THMDt2rpp	h[p] + thymd[p] <==> h[c] + thymd[c]	b2406	deletion
UDPGALM	[c] : udpgal --> udpgalfur	b2036	deletion
URIt2rpp	h[p] + uri[p] <==> h[c] + uri[c]	b2406	deletion
XTSNt2rpp	h[p] + xtsn[p] <==> h[c] + xtsn[c]	b2406	deletion
XYLabcpp	atp[c] + h2o[c] + xyl-D[p] --> adp[c] + h[c] + pi[c] + xyl-D[c]	(b3566 and b3567 and b3568)	deletion
ARAI	[c] : arab-L <==> rbl-L	b0062	deletion
ECA4OALpp	[p] : colipa + eca4und --> eca4colipa + h + udcpdp	b3622	deletion

GALT1	[c] : gicolipa + udpg --> gagicolipa + h + udp	b3628	deletion
GLCTR2	[c] : gagicolipa + udpg --> ggagicolipa + h + udp	b3627	deletion
GLCTR3	[c] : ggagicolipa + udpg --> gggagicolipa + h + udp	b3626	deletion
HEPK2	[c] : atp + hphhlipa --> adp + h + phphhlipa	b3625	deletion
HEPT4	[c] : adphep-LD + gggagicolipa --> adp + colipa + h	b3623	deletion
MOAT3C	[c] : ckdo + phphhlipa --> cmp + h + kphphhlipa	b3624	deletion
RHAT1	[c] : dtdprmn + kphphhlipa --> dtdp + h + icolipa	b3629	deletion
ASPt2_2pp	asp-L[p] + (2) h[p] --> asp-L[c] + (2) h[c]	b3528	turned off
FUMt2_2pp	fum[p] + (2) h[p] --> fum[c] + (2) h[c]	b3528	turned off
MALDt2_2pp	(2) h[p] + mal-D[p] --> (2) h[c] + mal-D[c]	b3528	turned off
MALt2_2pp	(2) h[p] + mal-L[p] --> (2) h[c] + mal-L[c]	b3528	turned off
OROTt2_2pp	(2) h[p] + orot[p] --> (2) h[c] + orot[c]	b3528	turned off
SUCCt2_2pp	(2) h[p] + succ[p] --> (2) h[c] + succ[c]	b3528	turned off
GLTPD	[c] : galt1p + nad <==> h + nadh + tag6p-D	b2091	turned off
GALTptspp	galt[p] + pep[c] --> galt1p[c] + pyr[c]	(b2094 and b2093 and b2092 and b2415 and b2416)	turned off
EX_succ(e)	[e] : sucr <==>		turned off
EX_asp_L(e)	[e] : asp-L <==>		turned off
EX_mal_D(e)	[e] : mal-D <==>		turned off
EX_mal_L(e)	[e] : mal-L <==>		turned off

Abbreviations and detailed information can be found in the previous paper reporing metabolic network model of *E. coli* K-12 MG1655 (Feist et al, 2007).
Eleven compounds were added and their names and chemical formulas are as follows: rbl-D (D-ribulose, C5H10O5), arab-D (D-arabinose, C5H10O5), 4hpa (4-hydroxyphenylacetate, C8H8O3), 34dhpl (3,4-dihydroxyphenylacetate, C8H8O4), 2h5cmmsa (2-hydroxy-5-carboxymethylmuconate semialdehyde, C8H8O6), 5c2hm (5-carboxymethyl-2-hydroxymuconate, C8H8O7), 5c2o3e (5-carboxy-2-oxohept-3-enedioate, C8H8O7), 2hh24d (2-hydroxyhepta-2,4-dienedioate, C7H8O5), 2o3e (2-oxohept-3-enedioate, C7H8O5), 24dhh2ed (2,4-dihydroxyhept-2-enedioate, C7H10O6), acgal6p (N-acetyl-D-galactosamine 6-phosphate, C8H14NO9P)

Table S5. Phenotypic differences of *E. coli* B REL606 and K-12 MG1655 in PM1 and PM2 and *in silico* predictions of cell growth on each carbon source.

PM1					Prediction	PM2					Prediction
**Well**	**Chemical**	**B***	**K-12***	**B/K-12[†]**	**B/K-12[‡]**	**Well**	**Chemical**	**B***	**K-12***	**B/K-12[†]**	**B/K-12[‡]**
A01	Negative control	0	0	–/–	–/–	A01	Background	0	0	–/–	–/–
A02	[a]**L-Arabinose**	6	182	–/++	–/+	A02	Chondroitin sulfate C	0	0	–/–	–/–
A03	N-Acetyl-D-glucosamine	297	198	++/++	+/+	A03	α-Cyclodextrin	0	0	–/–	–/–
A04	D-Saccharic acid	165	234	++/++	+/+	A04	β-Cyclodextrin	0	0	–/–	–/–
A05	[b]**Succinic acid**	45	215	+/++	–/+	A05	γ-Cyclodextrin	0	0	–/–	–/–
A06	D-Galactose	257	246	++/++	+/+	A06	Dextrin	296	251	++/++	–/–
A07	[b]**L-Aspartic acid**	67	232	+/++	–/+	A07	Gelatin	0	1	–/–	–/–
A08	[c]**L-Proline**	282	14	++/+	+/+	A08	Glycogen	4	0	–/–	–/–
A09	D-Alanine	228	227	++/++	+/+	A09	Inulin	0	3	–/–	–/–
A10	D-Trehalose	274	228	++/++	+/+	A10	Laminarin	0	0	–/–	–/–
A11	D-Mannose	276	209	++/++	+/+	A11	Mannan	0	0	–/–	–/–
A12	[d]**Dulcitol (galactitol)**	1	0	–/–	–/+	A12	Pectin	6	23	–/+	–/–
B01	D-Serine	262	215	++/++	+/+	B01	[e]**N-Acetyl-D-galactosamine**	294	1	++/–	+/–
B02	D-Sorbitol	255	224	++/++	+/+	B02	N-Acetyl-neuraminic acid	250	229	++/++	+/+
B03	Glycerol	217	213	++/++	+/+	B03	β-D-Allose	260	158	++/++	+/+
B04	[f]**L-Fucose**	0	232	–/++	–/+	B04	D-Amygdalin	0	0	–/–	–/–
B05	D-Glucuronic acid	298	263	++/++	+/+	B05	[g]**D-Arabinose**	249	13	++/–	+/–
B06	D-Gluconic acid	307	267	++/++	+/+	B06	D-Arabitol	0	0	–/–	–/–
B07	D,L-α-Glycerol- phosphate	176	151	++/++	+/+	B07	L-Arabitol	0	0	–/–	–/–
B08	[h]**D-Xylose**	8	196	–/++	+/+	B08	Arbutin	0	0	–/–	–/–
B09	L-Lactic acid	218	201	++/++	+/+	B09	2-Deoxy-D-ribose	6	6	–/–	–/–
B10	Formic acid	41	34	+/+	+/+	B10	I-Erythritol	0	0	–/–	–/–
B11	D-Mannitol	260	174	++/++	+/+	B11	D-Fucose	0	1	–/–	–/–
B12	L-Glutamic acid	251	5	++/–	+/+	B12	3-0-β-D-Galacto-pyranosyl-D-arabinose	224	164	++/++	–/–
C01	D-Glucose-6-phosphate	312	253	++/++	+/+	C01	Gentiobiose	0	29	–/+	–/–
C02	D-Galactonic acid-γ-lactone	268	234	++/++	+/+	C02	L-Glucose	0	0	–/–	–/–
C03	D,L-Malic acid	45	218	–/++	–/–	C03	Lactitol	0	0	–/–	–/–
C04	D-Ribose	300	294	++/++	+/+	C04	D-Melezitose	0	0	–/–	–/–
C05	Tween 20	9	0	–/–	–/–	C05	Maltitol	0	0	–/–	–/–
C06	L-Rhamnose	231	226	++/++	+/+	C06	α-Methyl-D-galactoside	0	0	–/–	–/–
C07	D-Fructose	287	166	++/++	+/+	C07	β-Methyl-D-galactoside	147	125	++/++	–/–
C08	Acetic acid	144	157	++/++	+/+	C08	3-Methyl glucose	0	0	–/–	–/–
C09	α-D-Glucose	279	169	++/++	+/+	C09	β-Methyl-D-glucuronic acid	234	136	++/++	–/–

C10	Maltose	257	191	++/++	+/+	C10	α-Methyl-D-mannoside	0	0	–/–	–/–
C11	D-Melibiose	260	192	++/++	+/+	C11	β-Methyl-D-xyloside	0	0	–/–	–/–
C12	Thymidine	276	190	++/++	+/+	C12	Palatinose	0	1	–/–	–/–
D01	L-Asparagine	70	179	+/++	+/+	D01	D-Raffinose	3	0	–/–	–/–
D02	D-Aspartic acid	0	0	–/–	–/–	D02	Salicin	1	0	–/–	–/–
D03	D-Glucosaminic acid	0	0	–/–	–/–	D03	Sedoheptulosan	0	0	–/–	–/–
D04	1,2-Propanediol	0	0	–/–	+/+	D04	L-Sorbose	0	0	–/–	–/–
D05	Tween 40	6	1	–/–	+/+	D05	Stachyose	0	0	–/–	–/–
D06	α-Keto-glutaric acid	40	213	+/++	+/+	D06	D-Tagatose	4	10	–/–	–/–
D07	α-Keto-butyric acid	1	148	–/++	–/–	D07	Turanose	0	0	–/–	–/–
D08	α-Methyl-D-galactoside	247	240	++/++	+/+	D08	Xylitol	0	0	–/–	–/–
D09	α-D-Lactose	263	168	++/++	+/+	D09	N-Acetyl-D-glucosaminitol	0	0	–/–	–/–
D10	Lactulose	141	160	++/++	–/–	D10	γ-Amino butyric acid	0	0	–/–	+/+
D11	Sucrose	0	0	–/–	+/+	D11	d-Amino valeric acid	0	0	–/–	–/–
D12	Uridine	252	221	++/++	+/+	D12	Butyric acid	0	2	–/–	+/+
E01	L-Glutamine	215	114	++/++	+/+	E01	Capric acid	1	6	–/–	+/+
E02	[b]**m-Tartaric acid**	0	150	–/++	–/–	E02	Caproic acid	3	0	–/–	+/+
E03	D-Glucose-1-phosphate	268	250	++/++	+/+	E03	Citraconic acid	0	0	–/–	–/–
E04	D-Fructose-6-phosphate	275	257	++/++	+/+	E04	Citramalic acid	0	0	–/–	–/–
E05	Tween 80	12	7	–/–	–/–	E05	D-Glucosamine	277	265	++/++	–/–
E06	α-Hydroxy glutaric acid-γ-lactone	1	0	–/–	–/–	E06	2-Hydroxybenzoic acid	0	0	–/–	–/–
E07	α-Hydroxy butyric acid	3	152	–/++	–/–	E07	4-Hydroxy benzoic acid sodium	0	0	–/–	–/–
E08	β-Methyl-D-glucoside	43	119	+/++	–/–	E08	β-Hydroxy butyric acid	0	0	–/–	–/–
E09	Adonitol	0	0	–/–	–/–	E09	γ-Hydroxy butyric acid	0	0	–/–	–/–
E10	Maltotriose	261	204	++/++	+/+	E10	2-Oxovaleric acid	0	0	–/–	–/–
E11	2'-Deoxy adenosine	260	201	++/++	+/+	E11	Itaconic acid	0	0	–/–	–/–
E12	Adenosine	255	186	++/++	+/+	E12	5-Keto-D-gluconic acid	19	76	+/+	+/+
F01	Glycyl-L-aspartic Acid	211	173	++/++	+/+	F01	D-Lactic acid methyl ester	235	200	++/++	–/–
F02	Citric acid	0	0	–/–	–/–	F02	Malonic acid	0	0	–/–	–/–
F03	m-Inositol	0	0	–/–	–/–	F03	Melibionic acid	240	64	++/+	–/–
F04	D-Threonine	4	0	–/–	+/+	F04	Oxolic acid	0	0	–/–	–/–
F05	[b]**Fumaric acid**	44	200	+/++	+/+	F05	Oxalomalic acid	0	0	–/–	–/–
F06	[b]**Bromo-succinic acid**	2	168	–/++	–/–	F06	Quinic acid	0	0	–/–	–/–
F07	Propionic acid	117	144	++/++	+/+	F07	D-Ribono-1,4-lactone	0	0	–/–	–/–
F08	Mucic acid	240	232	++/++	+/+	F08	Sebacic acid	0	0	–/–	–/–
F09	Glycolic acid	149	184	++/++	+/+	F09	Sorbic acid	1	0	–/–	–/–
F10	Glyoxylic acid	87	133	+/++	+/–	F10	Succinamic acid	0	0	–/–	–/–
F11	D-Cellobiose	0	0	–/–	–/–	F11	D-Tartaric acid	0	0	–/–	–/–

F12	Inosine	269	256	++/++	+/+	F12	L-Tartaric acid	0	0	–/–	+/+
G01	Glycyl-L-glutamic acid	207	165	++/++	–/–	G01	Acetamide	2	0	–/–	–/–
G02	Tricarballylic acid	1	0	–/–	–/–	G02	L-Alaninamide	69	50	+/+	+/+
G03	L-Serine	262	221	++/++	+/+	G03	N-Acetyl-L-glutamic acid	176	0	++/–	+/+
G04	L-Threonine	199	88	++/+	+/+	G04	L-Arginine	0	0	–/–	–/–
G05	L-Alanine	244	215	++/++	+/+	G05	Glycine	6	9	–/–	–/–
G06	L-Alanyl-glycine	250	227	++/++	–/–	G06	L-Histidine	0	0	–/–	–/–
G07	Acetoacetic acid	2	9	–/–	+/+	G07	L-Homoserine	0	0	–/–	–/–
G08	N-Acetyl-β-D-mannosamine	192	116	++/++	+/+	G08	Hydroxy-L-proline	0	0	–/–	–/–
G09	Mono methyl succinate	1	24	–/+	–/–	G09	L-Isoleucine	0	0	–/–	–/–
G10	Methyl pyruvate	241	196	++/++	–/–	G10	L-Leucine	0	0	–/–	–/–
G11	[b]**D-Malic acid**	2	209	–/++	–/+	G11	L-Lysine	0	0	–/–	–/–
G12	[b]**L-Malic acid**	68	200	+/++	–/+	G12	L-Methionine	1	0	–/–	–/–
H01	Glycyl-L-proline	224	223	++/++	–/–	H01	L-Ornithine	14	0	+/–	–/+
H02	[i]**p-Hydroxy phenyl acetic acid**	149	0	++/–	+/–	H02	L-Phenylalanine	0	0	–/–	–/–
H03	M-Hydroxy phenyl acetic acid	0	0	–/–	–/–	H03	L-Pyroglutamic acid	1	0	–/–	–/–
H04	Tyramine	0	0	–/–	–/–	H04	L-Valine	0	0	–/–	–/–
H05	D-Psicose	109	68	++/+	–/–	H05	D,L-Carnitine	0	0	–/–	–/–
H06	L-Lyxose	252	213	++/++	+/+	H06	Sec-butylamine	0	0	–/–	–/–
H07	Glucuronamide	15	1	–/–	–/–	H07	D.L-Octopamine	0	0	–/–	–/–
H08	Pyruvic acid	268	222	++/++	+/+	H08	Putrescine	2	0	–/–	+/+
H09	L-Galactonic acid-γ-lactone	2	227	–/++	+/+	H09	Dihydroxy acetone	108	161	++/++	+/+
H10	D-Galacturonic acid	306	254	++/++	+/+	H10	2,3-Butanediol	1	0	–/–	–/–
H11	Phenylethylamine	4	0	–/–	–/–	H11	2,3-Butanone	33	7	+/–	–/–
H12	2-Aminoethanol	13	0	–/–	+/+	H12	3-Hydroxy 2-butanone	16	0	+/–	–/–

*In each well, the unitless areas beneath growth-time curves for REL606 and MG1655 were calculated by OmniLog-PM software and were averaged for four PM tests. The areas were then subtracted from that of negative control.

†For the comparison with in silico cell growth, we considered cell growth, the experimental phenotype was considered as 'high growth' if the growth-area is over 100, 'low growth' if over 13, otherwise, the condition was assigned 'no growth'. The threshold for 'no growth' was chosen based on the standard deviation of the growth area where REL606 grew on L-arabinose.

‡*In silico* prediction of cell growth on each carbon source was made by flux balance analysis: '–' (no growth), '+' (growth)

[a]Inability of B to use L-arabinose is due to nonsynonymous substitutions in the L-arabinose isomerase gene (*araA*). Note that the mutation in *araA* was induced by MNNG (N-methyl-N'-nitro-N-nitrosoguanidine) in *E. coli* Bc251, the ancestor of REL606. Thus, inability of using L-arabinse is not a property of B strains in general (Jeong et al, 2009).

[b]Growth defects of B strain on C_4-dicarboxylates (bromo-succinic acid, succinic acid, fumaric acid, D-malic and L-malic acids, m-tartaric acid, and L-aspartic acid) may be attributed to inactivation of *dcuS* by a frameshift mutation. DcuS is a sensor kinase of the two-component system DcuSR that permits the utilization of external C_4-dicarboxylates (Golby et al, 1999).

[c]The growth defect of K-12 on L-proline seems to be caused by disruption of two putative transporters, *ycdN* adjacent to the proline symporter gene (*putP*) and

ygaY adjacent to the proline ABC transporter operon (*proVWX*).

[d]Genes for galactitol transport and metabolism were highly expressed at both the mRNA level (*gatYZC*) and the protein level (GatYZAB) in K-12. This can be because *gatR* which is a repressor of the *gat* genes is truncated in K-12 but intact in B.

[e]K-12 did not grow on the amino sugar N-acetyl-D-galactosamine because of a deletion of the *aga* cluster between *agaW* and *agaA* (Brinkkotter et al, 2000).

[f]Interruption of the L-fucolose-1-phosphate aldolase gene (*fucA*) by IS*1* insertion and loss of the L-fucose transporter gene (*fucP*) are responsible for the growth defect of B on L-fucose.

[g]Growth of B strain on D-arabinose can be explained by the existence of a specific gene cluster for utilization of D-arabinose (Elsinghorst & Mortlock, 1994).

[h]Insertional mutation of a gene (*xylG*) encoding a subunit of the D-xylose transporter evidently caused the growth defect of B on D-xylose.

[i]B genome has *hpa* cluster for degradation of 3- and 4- hydroxy phenyl acetic acid.

Supplementary References

Bradford MM (1976) A rapid and sensitive method for the quantitation of microgram quantities of protein utilizing the principle of protein-dye binding. *Anal Biochem* **72:** 248-254

Brinkkotter A, Kloss H, Alpert C, Lengeler JW (2000) Pathways for the utilization of N-acetyl-galactosamine and galactosamine in *Escherichia coli*. *Mol Microbiol* **37:** 125-135

Elsinghorst EA, Mortlock RP (1994) Molecular cloning of the *Escherichia coli* B L-fucose-D-arabinose gene cluster. *J Bacteriol* **176:** 7223-7232

Golby P, Davies S, Kelly DJ, Guest JR, Andrews SC (1999) Identification and characterization of a two-component sensor-kinase and response-regulator system (DcuS-DcuR) controlling gene expression in response to C_4-dicarboxylates in *Escherichia coli*. *J Bacteriol* **181:** 1238-1248

Laemmli UK (1970) Cleavage of structural proteins during the assembly of the head of bacteriophage T4. *Nature* **227:** 680-685

Moe D, Garbarsch C, Kirkeby S (1994) The protein effect on determination of DNA with Hoechst 33258. *J Biochem Biophys Methods* **28:** 263-276
